# A systematic approach identifies p53-DREAM pathway target genes associated with blood or brain abnormalities

**DOI:** 10.1242/dmm.050376

**Published:** 2023-10-11

**Authors:** Jeanne Rakotopare, Vincent Lejour, Carla Duval, Eliana Eldawra, Hugues Escoffier, Franck Toledo

**Affiliations:** ^1^ Genetics of Tumor Suppression, Institut Curie, Paris 75248 Cedex 05, France; ^2^ CNRS UMR3244, Paris 75005, France; ^3^ Sorbonne University, Paris 75005, France; ^4^ PSL Research University, Paris 75005, France; ^5^ Paris-Saclay University, Évry-Courcouronnes 91000, France

**Keywords:** p53, DREAM repressor complex, Bone marrow failure, Microcephaly, Cerebellar hypoplasia, Glioblastoma

## Abstract

p53 (encoded by *Trp53*) is a tumor suppressor, but mouse models have revealed that increased p53 activity may cause bone marrow failure, likely through dimerization partner, RB-like, E2F4/E2F5 and MuvB (DREAM) complex-mediated gene repression. Here, we designed a systematic approach to identify p53-DREAM pathway targets, the repression of which might contribute to abnormal hematopoiesis. We used Gene Ontology analysis to study transcriptomic changes associated with bone marrow cell differentiation, then chromatin immunoprecipitation-sequencing (ChIP-seq) data to identify DREAM-bound promoters. We next created positional frequency matrices to identify evolutionary conserved sequence elements potentially bound by DREAM. The same approach was developed to find p53-DREAM targets associated with brain abnormalities, also observed in mice with increased p53 activity. Putative DREAM-binding sites were found for 151 candidate target genes, of which 106 are mutated in a blood or brain genetic disorder. Twenty-one DREAM-binding sites were tested and found to impact gene expression in luciferase assays, to notably regulate genes mutated in dyskeratosis congenita (*Rtel1*), Fanconi anemia (*Fanca*), Diamond–Blackfan anemia (*Tsr2*), primary microcephaly [*Casc5* (or *Knl1*), *Ncaph* and *Wdr62*] and pontocerebellar hypoplasia (*Toe1*). These results provide clues on the role of the p53-DREAM pathway in regulating hematopoiesis and brain development, with implications for tumorigenesis.

## Teaser

**Editor's choice:** Putative DREAM-binding sites were identified for 151 genes associated with blood or brain abnormalities and mutation of 21 sites impacted gene expression, suggesting that p53-DREAM alterations contribute to bone marrow failure and glioblastoma cell phenotypes.

## INTRODUCTION

The dimerization partner, RB-like, E2F4/E2F5 and MuvB (DREAM) complex is a master coordinator of cell cycle-dependent gene expression that mediates gene repression in quiescent cells (Litovchick et al., 2007) and coordinates periodic gene expression in proliferating cells (Sadasivam and DeCaprio, 2013). Although p53 (encoded by *Trp53*) had been shown to repress transcription over 30 years ago (Ginsberg et al., 1991; Santhanam et al., 1991), its capacity to do so indirectly, via p21 (encoded by *Cdkn1a*) and the DREAM complex, emerged only progressively (Benson et al., 2014; Fischer et al., 2014a; Gottifredi et al., 2001; Löhr et al., 2003; Quaas et al., 2012; Taylor et al., 2001; Zhu et al., 2002). Meta-analyses first indicated that the p53-p21-DREAM pathway regulates G2/M cell cycle genes (Fischer et al., 2016a), then that it participates in the control of all cell cycle checkpoints (Engeland, 2018; Fischer et al., 2016b), and 85% of known targets of the p53-p21-RB pathway were recently proposed to be also regulated by p53-p21-DREAM signaling (Engeland, 2022). Furthermore, cells lacking LIN37, a subunit of the DREAM complex, demonstrated the functional impact of the p53-p21-DREAM (hereafter p53-DREAM) pathway in cell cycle regulation (Mages et al., 2017; Uxa et al., 2019).

However, the relative importance of this pathway remains to be fully appreciated, because multiple mechanisms were proposed to account for p53-mediated gene repression (Peuget and Selivanova, 2021). In fact, hundreds of genes were proposed to be regulated by the p53-DREAM pathway but, so far, only a few DREAM-binding sites (DBSs) were demonstrated experimentally, perhaps owing to the complexity of DREAM binding. The DREAM complex was initially reported to repress the transcription of genes for which the promoter sequences contain a bipartite binding motif called cell cycle-dependent element (CDE)/cell cycle gene homology region (CHR) (Müller and Engeland, 2010; Zwicker et al., 1995) (or E2F/CHR, Fischer et al., 2022b), with a GC-rich CDE that can be bound by E2F4 or E2F5, and an AT-rich CHR that can be bound by LIN54, the DNA-binding subunit of MuvB (Müller and Engeland, 2010; Zwicker et al., 1995). Later studies indicated that the DREAM complex can also bind promoters with a single E2F-binding site, a single CHR element, or a bipartite E2F/CHR-like element (CLE), and concluded that E2F and CHR elements are required for the regulation of G1/S and G2/M cell cycle genes, respectively (Fischer et al., 2016b; Müller et al., 2017). The TargetGeneRegulation (TGR) database of p53 and cell cycle genes (Fischer et al., 2022a) was reported to include putative DBSs for many human genes, based on separate genome-wide searches for 7-bp-long E2F or 5-bp-long CHR motifs, but the predicted sites were not tested experimentally. By contrast, positional frequency matrices (PFMs) designed to find bipartite DBSs were used to analyze only a few promoters, but their predictions were confirmed experimentally (Filipescu et al., 2017; Jaber et al., 2016).

Our interest in the p53-DREAM pathway stems from the analysis of a mouse model with increased p53 activity. We observed that mutant mice expressing p53^Δ31^, a truncated protein lacking 31 residues of the C-terminal domain, exhibited all the phenotypic traits associated with dyskeratosis congenita and its severe variant Hoyeraal–Hreidarsson syndrome, two bone marrow failure syndromes caused by defective telomere maintenance (Simeonova et al., 2013). Accordingly, *p53^Δ31/Δ31^* mice exhibited short telomeres and reduced expression of a few genes mutated in dyskeratosis congenita, notably *Rtel1*, the expression levels of which correlated with mouse survival (Simeonova et al., 2013). *p53^Δ31/Δ31^* cells also exhibited a reduced capacity to repair DNA interstrand cross-links, a typical feature of cells from patients with Fanconi anemia, another bone marrow failure syndrome (Jaber et al., 2016). This phenotype could be explained by reduced expression of several genes of the Fanconi anemia DNA repair pathway, including *Fancd2*, *Fanci* and *Rad51*, the promoters of which contain functionally relevant bipartite DBSs (Jaber et al., 2016). These findings appeared potentially relevant to human pathological processes, because p53 could also repress the homologous human genes (Jaber et al., 2016; Simeonova et al., 2013). In agreement with this, we later identified a germline missense mutation of *MDM4*, encoding a major negative regulator of p53, in a familial syndrome of neutropenia and defective telomere maintenance, and we could correlate p53 activation with decreased *RTEL1* expression and short telomeres in the most affected family member as well as in mice carrying the same *Mdm4* mutation (Toufektchan et al., 2020). Furthermore, two individuals carrying germline *TP53* mutations resulting in the expression of a truncated p53 protein lacking 32 C-terminal residues were recently reported (Toki et al., 2018). Consistent with our findings, these individuals exhibited increased p53 activity and short telomeres. Interestingly, however, they had a pure red cell aplasia resembling Diamond–Blackfan anemia, another bone marrow failure syndrome caused by ribosomal dysfunction – although the molecular mechanisms underlying impaired erythrocyte production in these patients remained unexplained (Toki et al., 2018). Taken together, these data indicated that germline p53 activation can cause a large spectrum of phenotypic traits found in patients with either dyskeratosis congenita, Fanconi anemia or Diamond–Blackfan anemia.

Our results in *p53^Δ31/Δ31^* mice led us to hypothesize that these phenotypic traits might partly result from gene repression mediated by the p53-DREAM pathway, which incited us to design a genome-wide approach relying on Gene Ontology (GO) analysis and bone marrow cell (BMC) differentiation to identify p53-DREAM targets related to hematopoiesis. Furthermore, mice and humans with germline increases in p53 activity can also exhibit microcephaly or cerebellar hypoplasia (Simeonova et al., 2013; Toki et al., 2018), and cerebellar hypoplasia can be observed in a subset of patients with bone marrow failure syndromes, including patients with Hoyeraal–Hreidarsson syndrome (Hoyeraal et al., 1970; Hreidarsson et al., 1988) or 27% of patients with Fanconi anemia (Fiesco-Roa et al., 2019). This led us to use the same strategy to search for candidate p53-DREAM target genes that might be involved in brain abnormalities.

With this study, we aimed to gain a better appreciation of the clinical relevance of the p53-DREAM pathway. We developed refined PFMs and identified bipartite DBSs in the promoters of 151 genes, many of which were not previously known to be DREAM targets. Most putative DBSs mapped at the level of chromatin immunoprecipitation-sequencing (ChIP-seq) peaks for DREAM subunits and near transcription start sites (TSSs), and a subset of the sites were tested with luciferase assays. Our study provides a resource of predicted DBSs for genes associated with blood and brain abnormalities, as well as a method that might be applied to analyze genes associated with other pathologies.

## RESULTS

### Candidate p53-DREAM target genes associated with blood abnormalities

**Fig. 1.**
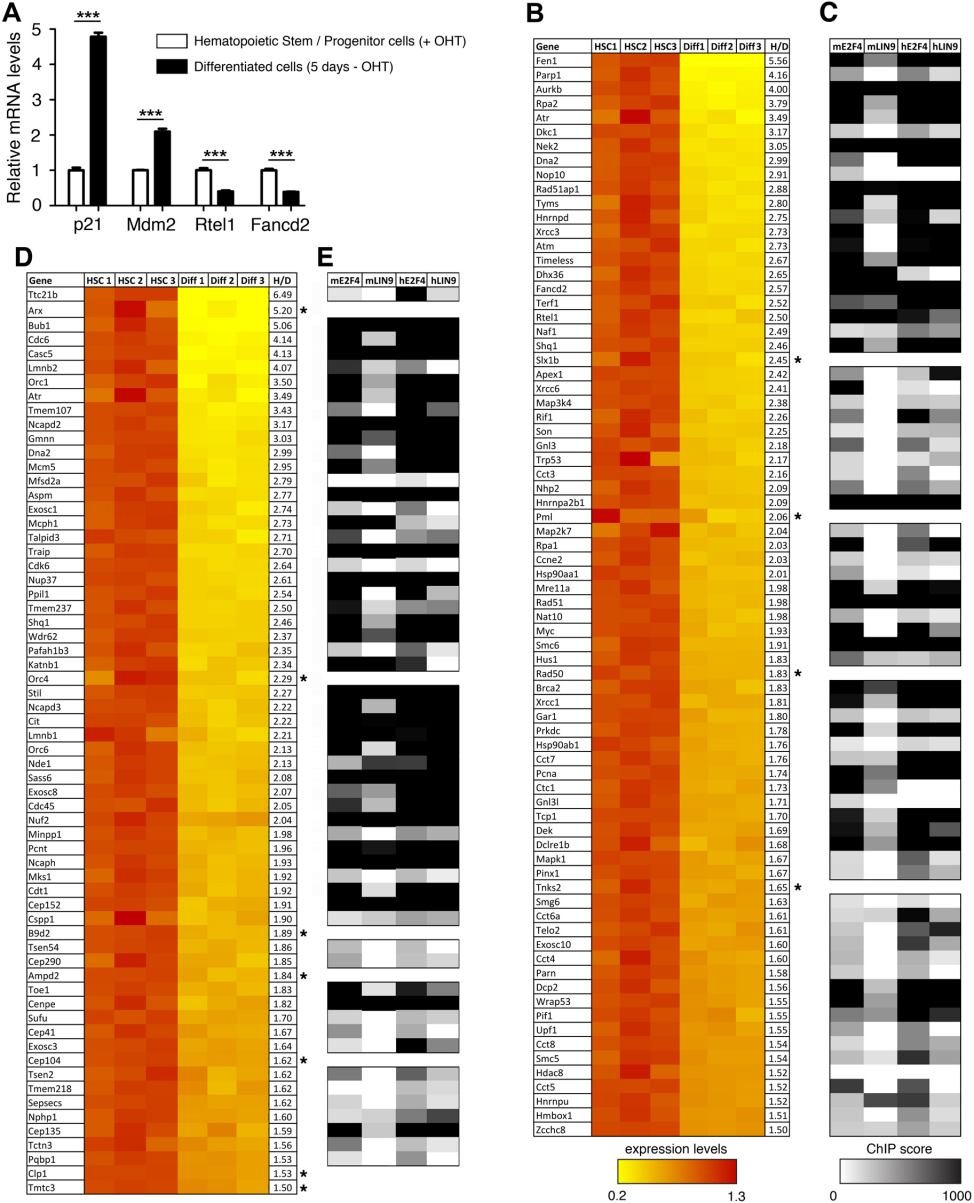
**Telomere-related or microcephaly-related genes downregulated upon bone marrow cell differentiation and their potential regulation by DREAM.** (A) The differentiation of Hoxa9-ER-expressing bone marrow cells (BMCs) correlates with p53 activation. Robust-multi average values for *p21* (*Cdkn1a*), *Mdm2*, *Rtel1* and *Fancd2* expression were extracted from transcriptome data of Hoxa9-ER-expressing hematopoietic stem and progenitor cells (HSCs) grown in the presence of tamoxifen (+OHT), or 5 days after tamoxifen withdrawal (−OHT) in differentiated cells (‘Diff’ in B,D). Average values (from triplicates) in cells with tamoxifen were given a value of 1. Means±s.e.m. are shown; ***P≤0.001 by two-tailed unpaired Student's *t*-test. (B) Telomere-related genes downregulated upon the differentiation of Hoxa9-ER-expressing BMCs. Expression values for 76 telomere-related genes from triplicates (indicated as HSC1-3 and Diff1-3) are shown; average values in cells with tamoxifen were given a value of 1. Genes are listed according to decreasing repression fold (‘H/D’, HSC/Diff). According to the TGR database, 72/76 genes are downregulated upon mouse and/or human p53 activation (the four exceptions are indicated with asterisks). (C) For each of the 72 p53-regulated genes in B, the highest chromatin immunoprecipitation (ChIP) scores of E2F4 or LIN9 binding in mouse (‘m’) or human (‘h’) cells are represented. Values shown are from ChIP-Atlas. (D) Genes associated with microcephaly syndromes and downregulated upon BMC differentiation. Expression values and ChIP scores represented as in B, for 64 genes associated with syndromes of microcephaly or cerebellar hypoplasia, of which 57 are downregulated by p53 (the exceptions are indicated with asterisks). (E) ChIP scores of E2F4 or LIN9 binding in mouse (‘m’) or human (‘h’) cells for the 57 microcephaly-related, p53-downregulated genes, represented as in C.

To investigate the role of the p53-DREAM pathway on the regulation of hematopoiesis, we exploited a transcriptomic approach in BMCs. The homeobox (Hox) family of transcription factors controls the proliferation, differentiation and self-renewal of hematopoietic stem cells. Notably, Hoxa9 is required for myeloid, erythroid and lymphoid hematopoiesis (Lawrence et al., 1997), and its overexpression causes hematopoietic stem cell expansion (Thorsteinsdottir et al., 2002). Muntean et al. (2010) generated a cellular model for Hoxa9 conditional expression. In this model, murine bone marrow stem and progenitor cells were immortalized by transduction with Hoxa9 fused with the estrogen receptor (ER) (Hoxa9-ER) in the presence of tamoxifen, and tamoxifen withdrawal led to their differentiation within 5 days. We observed that p53 activation correlated with cell differentiation in this system, because genes known to be transactivated by p53 (e.g. *Cdkn1a* and *Mdm2*) were induced, whereas genes repressed by p53 (e.g. *Rtel1* and *Fancd2*) were downregulated after tamoxifen withdrawal (Fig. 1A; see also Fig. S1 for additional examples of p53-regulated genes). Thus, to investigate the impact of p53 on telomere biology, we performed a GO analysis of the expression data obtained with this system (Gene Expression Omnibus GSE21299; Muntean et al., 2010), which relied on 45,101 microarray probes corresponding to 20,627 genes, of which 17,461 are associated with a GO term according to the Gene Ontology enRIchment anaLysis and visuaLizAtion tool (GOrilla) (Eden et al., 2009). We focused on genes downregulated at least 1.5-fold upon tamoxifen withdrawal. Such a downregulation was observed for 6880 probes, corresponding to 3631 genes associated with a GO term. According to the GOrilla tool, significant enrichment was observed for 13 GO terms related to telomere biology (Table 1). These 13 GO terms partially overlapped and corresponded to 68 different genes, including six genes (*Brca2*, *Dkc1*, *Gar1*, *Rad51*, *Rtel1* and *Terf1*) that we previously reported to be downregulated by p53 (Jaber et al., 2016; Simeonova et al., 2013). In addition, among the genes downregulated upon BMC differentiation, we noticed two genes (*Tyms* and *Zcchc8*) recently found to be associated with genetic disorders of telomere biology (Gable et al., 2019; Tummala et al., 2022), two genes (*Shq1* and *Son*) that might also impact telomere maintenance, and four p53-regulated genes (*Dek*, *Fancd2*, *Fen1* and *Timeless*) included in DNA repair-related GO terms but that also impact telomeres (Jaber et al., 2016). In sum, BMC differentiation correlated with the decreased expression of 76 genes that may impact telomere biology (Fig. 1B; Table S1). Consistent with the notion that BMC differentiation strongly correlates with p53 activation in this system, 72 of these 76 genes have negative p53 expression scores in the TGR database (Fischer et al., 2022a), which indicates that they were downregulated upon p53 activation in most experiments carried out in mouse and/or human cells (Fig. 1B; Table S1).

**Table 1.**
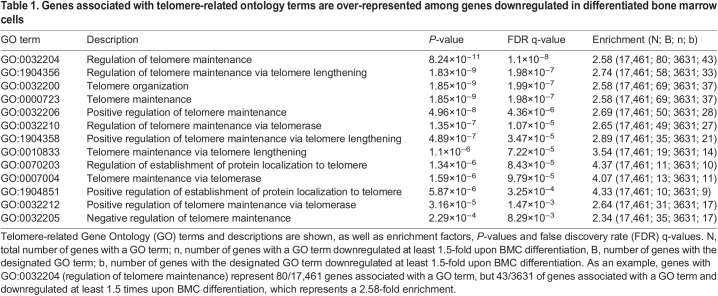
Genes associated with telomere-related ontology terms are over-represented among genes downregulated in differentiated bone marrow cells

We previously showed that p53 activation leads to increased binding of the E2F4 repressor at the promoters of four of these 72 telomere-related genes (*Brca2*, *Fancd2*, *Rad51* and *Rtel1*) (Jaber et al., 2016), which provided evidence that p53-mediated gene repression often occurs indirectly, through the recruitment of the E2F4-containing complex DREAM, often close to TSSs. Thus, we used ChIP-Atlas (Zou et al., 2022) to search for evidence of E2F4 binding at the promoters of the 72 telomere-related, p53-regulated candidate genes we had identified. The data compiled from 18 ChIP-seq experiments revealed E2F4 binding at 71 out of the 72 genes, in regions frequently overlapping TSSs (Fig. 1C; Table S2). To further identify candidate DREAM targets, we used ChIP-Atlas to search for evidence of MuvB binding. ChIP-Atlas does not have information on LIN54, the DNA-binding subunit of MuvB, so instead we analyzed the ChIP-seq data with antibodies against LIN9, another subunit of MuvB. The data compiled from four ChIP-seq experiments indicated LIN9 binding at 36 out of the 72 genes, at regions overlapping the regions bound by E2F4 (Fig. 1C; Table S2). Half the genes bound by E2F4 were not identified in LIN9 ChIP-seq experiments, which could suggest a regulation mediated by E2F4 independently of the DREAM complex. Alternatively, this might reflect technical limitations, resulting from the fact that LIN9 does not directly bind to DNA, or that the ChIP-seq data resulted from 18 experiments with antibodies against E2F4 but included only four experiments with antibodies against LIN9, or from qualitative differences between the antibodies used in the experiments. The repertoires of genes downregulated by the p53-DREAM pathway appear to be well conserved between humans and mice (Fischer, 2019), so we next analyzed ChIP-seq data from human cells. Evidence for binding by E2F4 was found for 68 out of the 72 homologous human genes, most often around the TSSs, and 59 of these genes were also bound by LIN9 (Fig. 1C; Table S3). Average ChIP-seq binding scores appeared slightly higher in human cells, particularly with antibodies against LIN9 (average score of 638 for 59 human genes, compared to 496 for 36 mouse genes). This suggests that antibodies against LIN9 might have been more efficient in precipitating the human LIN9 protein, so that the number of murine genes downregulated by p53-DREAM might have been underestimated owing to technical difficulties. In sum, 61 telomere-related genes were detected in ChIP assays with antibodies against E2F4 and LIN9 in at least one species (Fig. 1C), strengthening the notion that the p53-DREAM pathway plays a significant role in regulating telomere biology.

We previously reported that p53 can also downregulate many genes of the Fanconi anemia DNA repair pathway, a pathway implicated in the repair of DNA interstrand cross-links (Jaber et al., 2016). Accordingly, GOrilla revealed a significant enrichment for genes of the GO term ‘interstrand cross-link repair’ among the genes downregulated in murine differentiated BMCs (Table S4A). We found 55 genes downregulated upon BMC differentiation, encompassing genes mutated in Fanconi anemia, regulating the Fanconi DNA repair pathway and/or belonging to the Gorilla GO term ‘interstrand cross-link repair’, or to a recently proposed list of Fanconi anemia-related genes (Wang et al., 2021), including 52 genes downregulated by p53 according to the TGR database (Fig. S2A, Table S5). Out of these 52 genes, 12 are also known to impact telomere biology (*Atm*, *Atr*, *Brca2*, *Dclre1b*, *Fancd2*, *Fen1*, *Hus1*, *Rad51*, *Rad51ap1*, *Rpa2*, *Telo2* and *Xrcc3*). ChIP-seq experiments revealed E2F4 binding at 51 and LIN9 binding at 41 of the 52 genes (Fig. S2B), within regions frequently overlapping TSSs (Table S6). When we analyzed ChIP-seq data from human cells, evidence for binding by E2F4 was found for 51 of the 52 homologous genes, and 52/52 genes were bound by LIN9 (Fig. S2B), also at sequences frequently overlapping TSSs (Table S7).

Recent data suggested that some of the genes mutated in dyskeratosis congenita or Fanconi anemia may affect ribosomal function (Benyelles et al., 2019; Gueiderikh et al., 2021) and frameshift *TP53* mutations cause a pure red cell aplasia resembling Diamond–Blackfan anemia, together with relatively short telomeres (Toki et al., 2018). We thus also determined whether BMC differentiation altered the expression of genes involved in ribosome function. Indeed, among the genes downregulated at least 1.5-fold upon tamoxifen withdrawal, a significant enrichment was observed for 28 GO terms related to ribosome biology, rRNA biogenesis and maturation, and RNA polymerase I (Table S4B). These 28 GO terms partially overlapped and corresponded to 168 different genes, of which ten (*Dkc1*, *Exosc10*, *Gar1*, *Gnl3l*, *Naf1*, *Nat10*, *Nhp2*, *Nop10*, *Prkdc* and *Shq1*) are also known to impact telomere biology. Furthermore, we noticed three additional genes encoding subunits of RNA polymerase I (*Polr1d*, *Taf1c* and *Taf1d*) that were downregulated upon BMC differentiation, raising the total of candidates to 171 genes, of which 162 are downregulated by p53 according to the TGR database (Fig. S3, Table S8). E2F4 binding was found at 152 and LIN9 binding at 50 of the 162 murine genes, at sequences frequently overlapping TSSs (Fig. S3, Table S9). ChIP-seq data from human cells indicated binding by E2F4 for 153/162 and by LIN9 for 115/162 homologous genes, often within regions overlapping TSSs (Fig. S3, Table S10).

We next enquired whether genes mutated in other bone marrow disorders might be downregulated at least 1.5-fold upon BMC differentiation and found 17 candidate genes: *Ankrd26*, *Etv6* and *Mastl*, mutated in thrombocytopenia; *Pik3r1*, *Tcf3* and *Cd79b*, mutated in agammaglobulinemia; *Cdan1* and *Sec23b*, mutated in congenital dyserythropoietic anemia; *G6pc3* and *Gfi1*, mutated in severe congenital neutropenia; *Rbm8a*, mutated in thrombocytopenia absent radius syndrome; *Efl1*, mutated in Shwachman–Diamond syndrome type 2; *Rab27a*, mutated in Griscelli syndrome type 2; *Mtr*, mutated in homocystinuria megaloblastic anemia; *Mthfd1*, mutated in combined immunodeficiency and megaloblastic anemia with or without hyperhomocysteinemia; *Dnajc21*, mutated in bone marrow failure syndrome 3; and *Nuf2*, mutated in a bone marrow failure syndrome with microcephaly and renal hypoplasia. Out of these 17 genes, 15 were reported to be downregulated upon p53 activation (Fig. S4, Table S11). Out of the 15 genes, 14 were bound by E2F4 and six by LIN9 in murine cells (Fig. S4, Table S12), and 13 were bound by E2F4 and 12 by LIN9 in human cells (Fig. S4, Table S13).

We also used the Human Phenotype Ontology website (https://hpo.jax.org) (Köhler et al., 2021) to search for genes associated with abnormalities of blood and blood-forming tissues (ontology term #HP:0001871), and found that, out of a list of 1322 genes, 336 candidates were downregulated at least 1.5 times upon murine BMC differentiation, including 277 reported to be downregulated by p53 according to the TGR database (Fig. S5, Table S14). Out of these 277 genes, 243 were bound by E2F4 and 102 by LIN9, close to the TSSs in most cases (Fig. S5, Table S15). Out of the 277 human homologous genes, 245 were bound by E2F4 and 198 by LIN9 (Fig. S5, Table S16).

Together, the differentiation of BMCs correlated with the decreased expression of a total of 571 genes implicated in hematopoiesis, including 499 genes downregulated by p53 according to the TGR database (Table S17A,B; see also Fig. 3C for a summary of our approach). For 374 of these genes, E2F4 and LIN9 were found to bind at identical regions in at least one species (Table S17C). Furthermore, to focus on the best candidate p53-DREAM targets, we also considered the ChIP scores for E2F4 and LIN9 binding for each of the 374 genes. For each gene, we added the ChIP scores of E2F4 and LIN9 in both species, for a maximal value of 4000 (Table S18). Total ChIP scores ranged from 313 to 4000, and we noticed total ChIP scores of 656 and 720 for *Fbl* and *Dkc1*, respectively, two genes reported to be directly repressed by p53 binding (Marcel et al., 2013; Simeonova et al., 2013), and a score of 979 for *Exosc5*, a gene previously proposed to be regulated by DREAM (Fischer et al., 2016b). We thus considered the 269 genes with a total ChIP score ≥979 as the most likely candidate p53-DREAM targets (Tables S17D and S18).

**Fig. 2.**
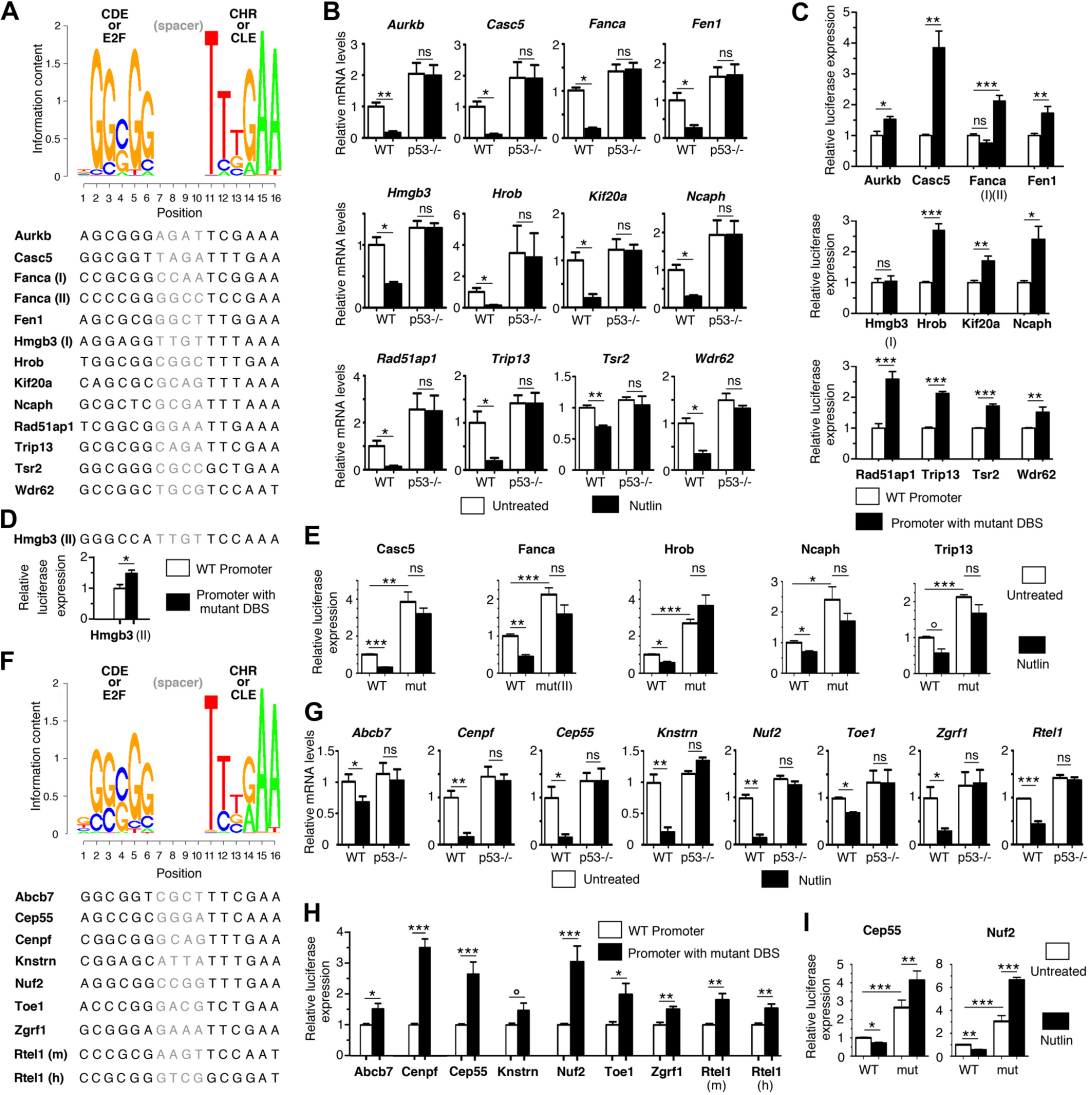
**Functional assays of putative DREAM-binding sites identified with positional frequency matrices.** (A) Representation of the positional frequency matrix (PFM) PFM10 and DNA sequences of 13 murine putative DREAM-binding sites (DBSs) identified with this matrix. PFM10 results from the DNA sequences of ten experimentally validated DBSs. Spacer DNA sequences between the GC-rich (CDE or E2F) and AT-rich (CHR or CLE) elements were not used to define the matrix (see Fig. S7A for details). (B) In mouse embryonic fibroblasts (MEFs), p53 activation led to the downregulation of the 12 tested genes. mRNAs from wild-type (WT) and *p53^−/−^* MEFs, treated with or without 10 mM Nutlin (an MDM2 antagonist) for 24 h, were quantified using real-time PCR, normalized to control mRNA levels, then the amount in WT untreated cells was assigned a value of 1. Means±s.e.m. from three independent experiments are shown. Similar results for *Fanca* and *Fen1* were reported previously (Jaber et al., 2016). (C) Luciferase assays of the putative DBSs reported in A. For each candidate gene, a 1-1.5 kb fragment containing WT promoter sequences or the same promoter with point mutations affecting the DBS was cloned upstream of a luciferase reporter gene (tested DBSs were from the species with best PFM score). WT or mutant luciferase reporter plasmids were transfected into NIH3T3 cells, luciferase activity was measured 24 h after transfection, and activity with the WT construct was assigned a value of 1. Results are from ≥4 values and ≥2 independent cellular experiments for each tested plasmid. (D) DNA sequence of an alternate putative DBS at the *Hmgb3* promoter and its validation by luciferase assays, carried out as above. (E) Mutating the DBSs in the *Casc5*, *Fanca*, *Hrob*, *Ncaph* or *Trip13* promoters abrogated their p53-dependent repression. WT or mutant (mut) luciferase plasmids were transfected into NIH3T3 cells, treated with or without Nutlin, then luciferase activity was measured after 24 h. Results are from ≥4 values and ≥2 independent cellular experiments for each tested plasmid. (F) Representation of PFM22 and DNA sequences of nine tested DBSs. PFM22 results from ten DBSs validated in previous reports and 12 DBSs tested in the present study, with spacer DNA sequences not used to define the matrix (see Fig. S7B for details). (G) In MEFs, p53 activation led to the downregulation of the eight tested genes. mRNAs from WT and *p53^−/−^* MEFs were treated and analyzed as in B. Similar results for *Rtel1* were reported previously (Simeonova et al., 2013). (H) Luciferase assays of the putative DBSs reported in F, performed as in C. (I) Mutating the DBSs in the *Cep55* or *Nuf2* promoter abrogated their p53-dependent repression. Luciferase assays were performed as in E. In A,C-E, ‘I’ and ‘II’ represent putative DBSs. All data show the mean±s.e.m. ns, not significant; °*P*≤0.07; *P<0.05; **P<0.01; ***P<0.001 (two-tailed paired or unpaired Student's *t*-test).

**Fig. 3.**
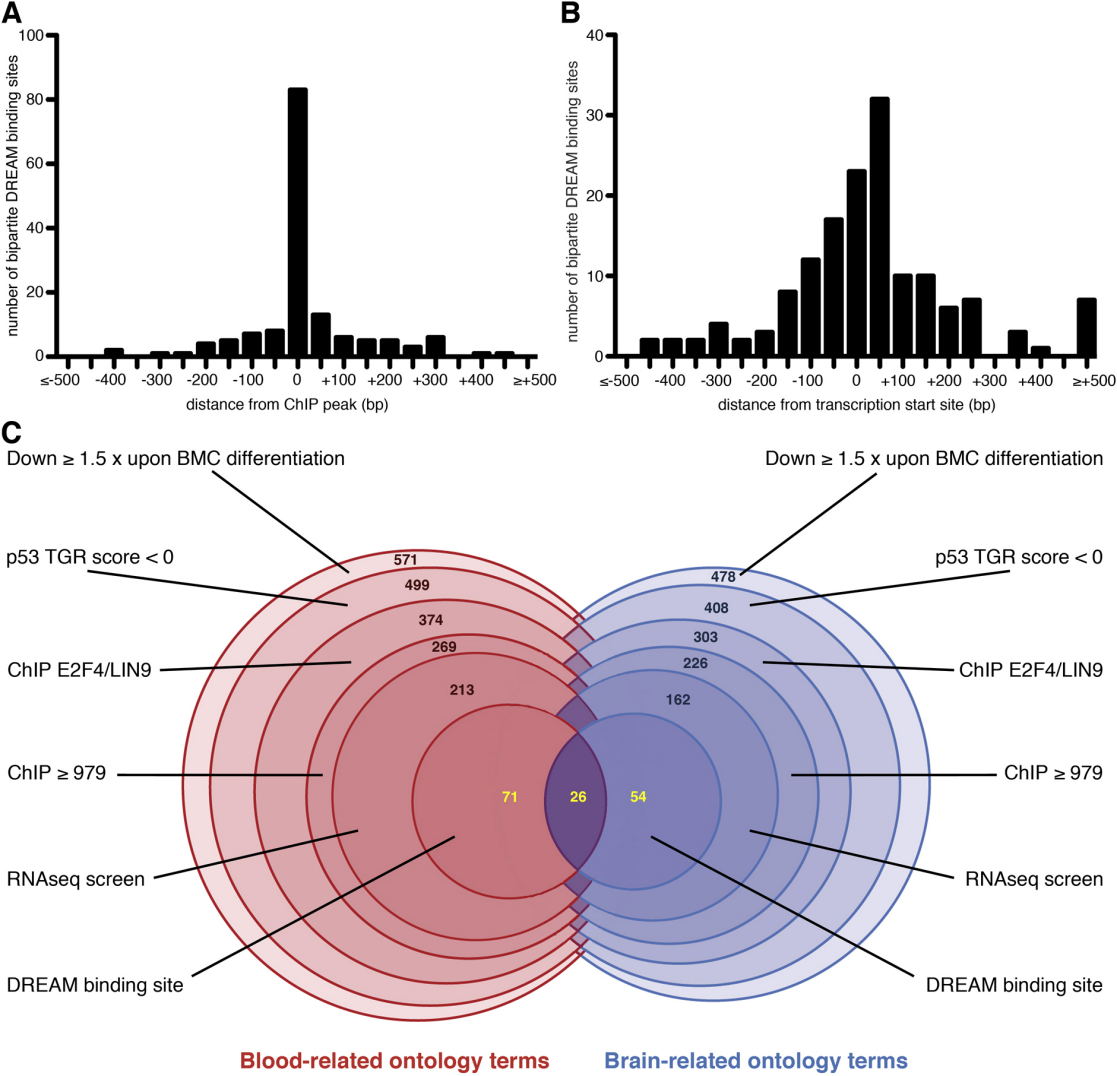
**Mapping of putative DBSs and summary of our systematic approach.** (A) Mapping of the putative DBSs relative to the ChIP peaks of E2F4 and/or LIN9 binding in 50 bp windows, for the 151 genes listed in Table 2. (B) Mapping of the putative DBSs relative to the transcription start sites of the 151 genes, in 50 bp windows. (C) Venn-like diagram of our systematic approach. Numbers in black indicate, at each step of the approach, genes related either to blood-related ontology terms or to brain-related ontology terms, which were analyzed separately (see text for details). In the last step, blood- and brain-related candidate genes were analyzed together to search for DBSs, and numbers in yellow indicate genes related to blood-related terms only, brain-related terms only, or both blood- and brain-related terms. Detailed lists of genes can be found in Tables S17 and S26.

**Table 2.**
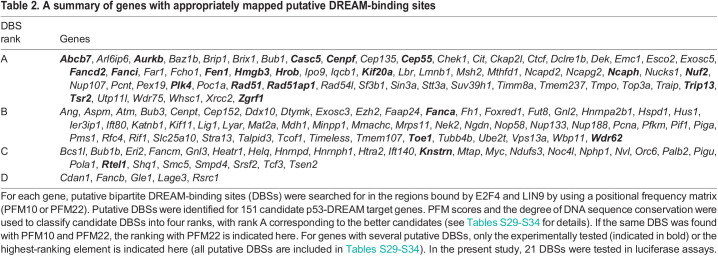
A summary of genes with appropriately mapped putative DREAM-binding sites

To estimate the relevance of this list of 269 candidates, we analyzed the dataset GSE171697, which includes RNA-sequencing (RNAseq) data from hematopoietic stem cells of unirradiated *p53* knockout (KO) mice, unirradiated wild-type (WT) mice or irradiated WT mice (Tung et al., 2021). We also analyzed GSE204924, with RNAseq data from splenic cells of irradiated *p53^Δ24/−^* or *p53^+/−^* mice (Resnick-Silverman et al., 2023). The public data from this dataset, although incomplete, appeared interesting because *p53^Δ24^* is a mouse model prone to bone marrow failure (Hamard et al., 2013) and the spleen is a hematopoietic organ in mice (Iseki et al., 2008). As expected, increased p53 activity correlated with an average increase in expression for 15 genes known to be transactivated by p53, with 13/15 genes upregulated at least 1.5-fold (Table S19). By contrast, only 56/269 candidate p53-DREAM target genes appeared to be upregulated in cells with increased p53 activity (Table S19). These 56 genes were considered poor candidate p53-DREAM targets and removed from further analyses, leading to a list of 213 candidate p53-DREAM targets related to blood abnormalities (Tables S17E and S19).

### Candidate p53-DREAM target genes associated with brain abnormalities

We next searched for p53-DREAM target genes for which altered expression might contribute to brain abnormalities. Many genes in which mutations cause microcephaly or cerebellar hypoplasia encode proteins implicated in fundamental processes common to all somatic cells (e.g. chromosome condensation, mitotic spindle activity or tRNA splicing). We thus reasoned that BMC differentiation data could also be exploited to search for genes downregulated upon p53 activation and implicated in these diseases. To test this, we used a candidate approach and searched for genes that might be regulated by the p53-DREAM pathway among: the 30 genes mutated in primary microcephaly; 23 genes mutated in pontocerebellar hypoplasia; 39 genes mutated in hypoplasia of the cerebellar vermis (Joubert syndrome); 18 genes mutated in syndromes combining microcephaly and dwarfism (Seckel syndrome, Meier–Gorlin syndrome or microcephalic osteodysplastic primordial dwarfism); 12 genes mutated in lissencephaly (often associated with microcephaly); *Nuf2*, mutated in a bone marrow failure syndrome with microcephaly and renal hypoplasia; *Pafah1b3*, truncated in a case of brain atrophy; *Pqbp1*, mutated in Renpenning syndrome (an X-linked syndrome of microcephaly); and *Shq1*, mutated in a syndrome with cerebellar hypoplasia, dystonia and seizures; for a total of 126 candidate genes. Downregulation of gene expression of at least 1.5-fold upon BMC differentiation was found for 64 of these candidates, including 57 reported to be downregulated upon p53 activation according to the TGR database (Fig. 1D; Table S20). Out of the 57 genes, 55 were bound by E2F4 and 36 by LIN9, within regions overlapping the TSSs in most cases (Fig. 1E; Table S21). Out of the 57 human homologs, all were bound by E2F4 and 49 by LIN9 (Fig. 1E; Table S22).

We next searched the Human Phenotype Ontology website for genes associated with microcephaly or cerebellar hypoplasia (ontology terms HP:0000252 and HP:0007360) and found that, out of a list of 1430 genes, 474 candidates were downregulated at least 1.5 times upon murine BMC differentiation, including 404 reported to be downregulated upon p53 activation (Fig. S6, Table S23). Out of these 404 genes, 354 were bound by E2F4 and 153 by LIN9, in regions overlapping TSSs in most cases (Fig. S6, Table S24). Out of the 404 human homologous genes, 371 were bound by E2F4 and 292 by LIN9 (Fig. S6, Table S25).

In sum, the differentiation of BMCs correlated with the decreased expression of 478 genes implicated in microcephaly or cerebellar hypoplasia, including 408 downregulated upon p53 activation according to the TGR database (Table S26A,B). For 303 of these genes, E2F4 and LIN9 were found to bind at identical regions in at least one species (Table S26C). Furthermore, total ChIP scores ≥979 were found for 226 of the 303 genes, which appeared as better candidate p53-DREAM targets (Tables S26D and S27).

To estimate the relevance of this list of 226 candidates, we analyzed the datasets GSE78711 and GSE80434, containing RNAseq data from human cortical neural progenitors infected by the Zika virus (ZIKV) or mock infected, because ZIKV was shown to cause p53 activation in cortical neural progenitors and microcephaly (Tang et al., 2016; Zhang et al., 2016). Accordingly, most genes (12/16) known to be transactivated by p53 were upregulated in ZIKV-infected cells (Table S28). By contrast, only 64/226 candidate p53-DREAM target genes appeared to be upregulated in ZIKV-infected cells (Table S28). These 64 genes were considered poor candidate p53-DREAM targets and removed from further analyses, leading to a list of 162 candidate p53-DREAM targets related to brain abnormalities (Tables S26E and S28). Importantly, out of the 162 microcephaly-related candidate genes identified (Table S26E), 58 also belonged to the list of 213 genes associated with abnormal hematopoiesis (Table S17E), consistent with the notion that deregulation of the p53-DREAM pathway might be involved in both pathological processes. In sum, we identified 317 genes (213+162−58) downregulated upon BMC differentiation and p53 activation, bound by E2F4 and LIN9 in at least one species, with total ChIP scores ≥979, that appeared as better candidate p53-DREAM targets after analyzing appropriate RNAseq data (Tables S17 and S26).

### Identification of DBSs in candidate target gene promoters

We aimed to obtain further evidence of DREAM-mediated regulation for the better candidates by searching for putative DREAM-binding sites (DBSs) within the regions bound by E2F4 and/or LIN9. Among the 213 candidate genes associated with blood abnormalities found here, we previously identified well-conserved bipartite DBSs, functional in both mouse and human species, for *Fancd2*, *Fanci* and *Rad51* (Jaber et al., 2016). Accordingly, we next used DNA sequence conservation as a criterion to identify the best putative bipartite DBSs within the regions bound by E2F4 and LIN9. We created a PFM based on ten functionally demonstrated murine DBSs (PFM10, Fig. 2A; Fig. S7A) and used PWMScan (Ambrosini et al., 2018) to search for putative DBSs in both mouse (mm10) and human (hg38) genomes, with a *P*-value threshold of 10^−3^. Based on our previous data with Fanc genes (Jaber et al., 2016), we focused our search on DBSs in the same orientation as the gene transcripts. This led us to identify putative DBSs for 55 genes associated with blood abnormalities (Table S29). The PFM score and degree of DNA sequence conservation were used to classify candidate DBSs into four categories: ranks A-C for DBSs with positive PFM scores and zero or one (rank A), two or three (rank B), or four (rank C) mismatches between mouse and human sequences at positions 2-6 or 11-16 of the consensus sequence, and rank D for DBSs with negative PFM scores and zero or one mismatches (see Table S29 for details). Likewise, we used PWMScan with PFM10 and sequence conservation to identify putative DBSs in promoters of the 162 genes associated with brain abnormalities. DBSs with various PFM scores and degrees of DNA sequence conservation were identified for 52 genes, of which 15 were also associated with blood abnormalities (Table S30).

A fraction of the putative DBSs identified with this approach were already shown to be functional in previous reports by using luciferase assays. This is the case for at least one of the two overlapping DBSs at the human *AURKB* promoter (Kimura et al., 2004), the murine DBS at the *Plk4* promoter (Fischer et al., 2014b), and for both the murine and human DBSs at the *Fancd2*, *Fanci* and *Rad51* promoters (Jaber et al., 2016) (Tables S29 and S30). Although testing the functionality of all the putative DBSs was beyond the scope of our study, we aimed to test the validity of our predictions by performing luciferase assays on a subset of the elements. We tested the putative DBSs of the following 12 genes: *Hmgb3*, *Hrob*, *Ncaph* and *Trip13*, containing putative DBSs of rank A; *Aurkb*, containing two overlapping DBSs of ranks A and B (shifted by only one nucleotide and thus similar to a single DBS); *Fanca*, containing two non-overlapping putative DBSs of ranks B and C; *Wdr62*, containing a putative DBS of rank B; and *Casc5*, *Fen1*, *Kif20a*, *Rad51ap1* and *Tsr2*, containing putative DBSs of rank D (Fig. 2A; Tables S29 and S30). According to GO analysis, these genes are associated with either abnormal hematopoiesis (*Aurkb*, *Fen1*, *Hrob*, *Kif20a*, *Rad51ap1* and *Tsr2*) or microcephaly (*Casc5*, *Hmgb3*, *Ncaph* and *Wdr62*), or both (*Fanca* and *Trip13*). For genes associated with abnormal hematopoiesis, we first verified that their expression was decreased in BMCs from *p53^Δ31/Δ31^* mice, prone to bone marrow failure, compared to their expression in WT BMCs (Fig. S8). We next determined, as a prerequisite to luciferase assays, that the expression of all tested genes, as well as their p53-mediated repression, could be observed in mouse embryonic fibroblasts (MEFs), because luciferase assays rely on transfections into the MEF cell line NIH3T3 (Fig. 2B). We cloned the promoters of the candidate targets upstream of a luciferase reporter gene, then introduced point mutations specific to the putative DBS element to abolish its potential function. In these experiments, the DBS for murine *Aurkb* served as a positive control because of its high sequence conservation with the DBS shown to be functional in the homologous human gene (Kimura et al., 2004). Consistent with its expected role in gene repression, the mutation of the DBS for murine *Aurkb* led to increased luciferase expression (Fig. 2C). A similar result was obtained with DBSs for ten of the 11 other tested genes [*Casc5*, *Fanca* (putative DBS II), *Fen1*, *Hrob*, *Kif20a*, *Ncaph*, *Rad51ap1*, *Trip13*, *Tsr2* and *Wdr62*; Fig. 2C]. For *Hmgb3*, however, the putative DBS element did not appear to be functional in luciferase assays. We reasoned that an improved PFM that would include the 11 additional DBSs we tested might lead to the identification of a proper DBS for this gene. Indeed, the second matrix (PFM21) suggested a new putative DBS at the *Hmgb3* promoter, the mutation of which affected gene expression in luciferase assays (Fig. 2D; Table S30). Of note, NIH3T3 cells exhibited an attenuated p53 pathway compared to that in primary WT MEFs (Fig. S9A). This facilitated cell survival after lipofections required in luciferase assays but led to decreased p53-DREAM-mediated gene repression (Fig. S9B). Under these experimental conditions, p53 activation in transfected NIH3T3 cells led to the robust repression (>1.4-fold) of five WT promoters (for *Casc5*, *Fanca*, *Hrob*, *Ncaph* and *Trip13*) cloned upstream of the luciferase reporter gene. Importantly, the p53-mediated repression of these five promoters was abrogated by mutating the identified DBSs (Fig. 2E), providing direct evidence of the functional relevance of DBSs identified with our PFM.

These experiments indicated that we could identify sites impacting luciferase expression for 12/12 tested genes, and we next integrated these sites into a third PFM (PFM22) used in all further analyses (Fig. 2F; Fig. S7B). We used PWMscan with PFM22 and a *P*-value threshold of 10^−3^ to reanalyze the genes for which putative DBSs had been suggested by using PFM10 (Tables S31 and S32). We reasoned that good candidate DBSs identified with PFM10 were likely to be found again with PFM22: this was verified for 45/55 hematopoiesis-related genes and 37/52 microcephaly-related genes. Furthermore, alternative DBSs (often with better scores) were suggested with PFM22 for 7/55 hematopoiesis-related genes and 9/52 microcephaly-related genes. For a few genes (e.g. *Cdan1* and *Gle1*), the putative DBSs identified with PFM10 were not detected with PFM22 and appeared as potentially weaker candidates. We also considered the converse situation – that for some genes for which no DBSs had been suggested with PFM10, it might be possible to find putative DBSs with PFM22. Indeed, the use of PFM22 made it possible to find putative DBSs for 57 additional targets (Table S33).

Thus, out of 317 genes associated with blood and/or brain abnormalities that appeared as potential DREAM targets, we found 149 genes containing at least one appropriately mapped putative bipartite DBS, in the same orientation as transcription, and with partial or complete DNA sequence conservation between human and mouse. These genes include *Abcb7*, *Cep55*, *Cenpf*, *Knstrn*, *Nuf2*, *Toe1* and *Zgrf1*, for which putative DBSs were also tested in luciferase assays (Fig. 2G-I). As for the genes for which no DBSs were suggested with our PFMs, we hypothesized that they might be regulated via DBSs not fulfilling our criteria. For example, a CDE/CHR was shown to regulate the expression of murine *Ccnb2*, but only the CHR element was conserved in the homologous human gene (Lange-zu Dohna et al., 2000; Wasner et al., 2003). Similarly, we identified a bipartite DBS in the murine *Rtel1* promoter for which only the CDE (E2F) element was conserved in the human homolog, and a DBS in the human *RTEL1* promoter for which only the CDE (E2F) element was conserved in the murine homolog (Fig. 2F-H; Table S34). Similar cases, i.e. putative DBSs with positive scores in one species and perfect conservation of either the CDE (E2F) at positions 2-6 or the CHR (CLE) at positions 11-16, were found for five other genes (Table S34). Of note, because these sites correspond to DBSs with positive scores and limited DNA sequence conservation, most had already been detected as sites of rank C (at the promoters of *Helq*, *Htra2*, *Ndufs3* and *Smc5*). Accordingly, DBSs with positive PFM22 scores and either four mismatches anywhere in the DBS or more mismatches but affecting only the CDE (E2F) or only the CHR (CLE) were together classified as rank C sites. Finally, our PFMs were designed to identify bipartite DBSs with a CDE (E2F) motif separated from a CHR (CLE) motif by a spacer of 4 bp. Presumably, candidate DREAM targets for which no DBS was identified with these PFMs might be bound by DREAM either via a bipartite site with spacer sequences of a different length, or by a single E2F or a single CHR motif, as previously proposed (Fischer et al., 2016b; Müller et al., 2017).

Table 2 summarizes our results: putative DBSs were identified in the promoters of 151 genes, including 97 genes associated with blood-related ontology terms and 80 with brain-related ontology terms (Tables S17F and S26F). Consistent with a functional relevance of the predicted DBSs, most sites co-mapped with peaks of E2F4 and/or LIN9 binding (Fig. 3A). At the 151 promoters, 83 putative DBSs mapped in a 50-bp-long window centered on ChIP peaks (Fig. 3A), whereas the frequency of putative DBSs per 50-bp-long windows was 4×10^−4^ over the entire human genome, indicating a 1300-fold enrichment of DBSs at ChIP peaks. This significant enrichment (f=3×10^−239^ in a hypergeometric test) is most likely underestimated because mouse-human DNA sequence conservations were not determined for putative DBSs over the full genome. In addition, it was proposed that DREAM primarily associates with nucleosomes near the TSSs of its targets (Asthana et al., 2022), and the distribution of predicted DBSs was consistent with this notion (Fig. 3B). Altogether, the differentiation of BMCs correlated with the downregulation of 571 genes associated with blood-related ontology terms and 478 genes associated with brain-related ontology terms (Fig. 3C), for a total of 883 genes (166 genes being associated with both blood- and brain-related terms, see Tables S17A and S26A). Out of these 883 genes, 760 (499+408–147) were reported to be downregulated by p53 (Fig. 3C; Tables S17B and S26B). Among those genes, our systematic approach identified 317 likely p53-DREAM targets, and our PFMs appeared as powerful tools to predict DBSs for about half of these target genes (Fig. 3C; Tables S17E,F and S26E,F).

## DISCUSSION

The capacity of p53 to activate the transcription of many targets, including genes important for cell cycle arrest (e.g. *CDKN1A*), apoptosis [e.g. *BAX* and *PUMA* (also known as *BBC3*)] or cellular metabolism (e.g. *TIGAR*), has been recognized for decades. In contrast, the potential importance of p53-dependent transcriptional repression has only emerged in recent years, in part because the mechanisms underlying p53-mediated repression remained controversial. In this report, we provide evidence for a general role of the p53-DREAM pathway in regulating genes associated with blood and/or brain abnormalities. We identified 317 potential p53-DREAM targets, i.e. genes with a decreased expression associated with murine BMC differentiation and p53 activation, and the promoter sequences of which can be significantly bound by two subunits of the DREAM complex in mouse and/or human cells. Among these potential targets, we identified putative DBSs in the promoter of 151 genes, and the mutation of a subset of these binding sites affected gene expression in luciferase assays.

Our approach has methodological similarities with the approaches described by Fischer et al., who first provided evidence that p53 often represses transcription indirectly via the DREAM or RB/E2F pathways (Fischer et al., 2014a), then reported lists of most likely candidate p53-DREAM targets – a first list of 210 genes, most of which were regulators of the G2/M phases of the cell cycle (Fischer et al., 2016a), then a list of 971 G1/S- or G2/M-phase cell cycle genes (Fischer et al., 2016b). Here, we found 883 genes related to blood- and/or brain-related ontology terms downregulated upon BMC differentiation, of which 760 were reported to be downregulated by p53. E2F4 and LIN9 were found to bind at the promoters of at least 317 genes downregulated by p53, consistent with a major role of the DREAM complex in p53-mediated repression. Interestingly, however, out of the 151 p53-DREAM targets with putative DBSs that we identified, only 30 were in the first list of 210 candidate DREAM targets, and 95 in the second list of 971 candidate DREAM targets reported by Fischer et al. The differences in p53-DREAM target repertoires might result in part from the fact that Fischer et al. mostly analyzed human fibroblasts treated with doxorubicin or nutlin, whereas we analyzed the effects of murine BMC differentiation. Interestingly, we identified *Brip1* as a p53-DREAM target gene, downregulated upon the differentiation of BMCs (Fig. S2) and in ZIKV-infected neural progenitors (Table S28), but not upon irradiation of hematopoietic stem cells (Table S19), consistent with the notion that different cellular responses might regulate partially distinct repertoires of DREAM targets. In addition, compared to Fischer et al., our systematic use of pathology-related GO likely created a sharper focus on clinically relevant target genes. In support of this, the list of 210 genes by Fischer et al. (2016a) included only one gene mutated in Fanconi anemia (*Fancb*) and no genes mutated in dyskeratosis congenita, whereas in a previous study with mouse fibroblasts focusing on these bone marrow failure syndromes (Jaber et al., 2016), we found evidence for the p53-mediated repression of eight clinically relevant genes that belong to our current list of 151 targets (*Fanca*, *Fancb*, *Fancd2*, *Fanci*, *Palb2*, *Rad51*, *Rtel1* and *Ube2t*).

Cells with a knockout of LIN37, a subunit of the DREAM complex, can also be used to identify potential DREAM targets (Mages et al., 2017; Uxa et al., 2019). For example, Mages et al. (2017) used CRISPR-Cas9 to generate *Lin37* KO murine cells, which were then rescued by an episomal *Lin37* expression vector, and *Lin37* KO and *Lin37*-rescued cells were compared by RNAseq analyses. Our list of 151 genes overlaps only partially with the list of candidate DREAM targets obtained with this approach, with 51/151 genes reported to be downregulated in *Lin37*-rescued cells (Mages et al., 2017). To better evaluate the reasons for this partial overlap, we extracted the RNAseq data from *Lin37* KO and *Lin37*-rescued cells and focused on the 151 genes in our list. For the 51 genes that Mages et al. (2017) reported as being downregulated in *Lin37*-rescued cells, an average downregulation of 14.8-fold was observed (Fig. S10, Table S35). Furthermore, when each gene was tested individually, a downregulation was observed in all cases, statistically significant for 47 genes and with a *P*-value between 0.05 and 0.08 for the remaining four genes (Table S35). By contrast, for the 100 genes not previously reported to be downregulated in *Lin37*-rescued cells, an average downregulation of 4.7-fold was observed (Fig. S10, Table S35) and each gene appeared to be downregulated, but this downregulation was statistically significant for only 35/100 genes and *P*-values between 0.05 and 0.08 were found for 23/100 other genes (Table S35). These comparisons suggest that, for the additional 100 genes, a more subtle decrease in expression, together with experimental variations, might have prevented identification of their DREAM-mediated regulation in *Lin37*-rescued cells.

Importantly, our approach integrated evolutive PFMs to identify putative bipartite DBSs in the promoters of candidate target genes. Most putative DBSs co-mapped with ChIP peaks for DREAM subunits and TSSs, and most DBSs tested experimentally were found to affect gene expression in luciferase assays, suggesting reliable DBS predictions. The TGR database of p53 and cell cycle genes was reported to include putative DBSs for human genes, based on separate genome-wide searches for 7-bp-long E2F or 5-bp-long CHR motifs (Fischer et al., 2022a). We analyzed the predictions of the TGR database for the 151 genes for which we had found putative bipartite DBSs. A total of 342 E2F binding sites were reported at the promoters of these genes, but only 64 CHR motifs were reported. The similarities between the predicted E2F or CHR sites from the TGR database and our predicted bipartite DBSs appeared rather limited: only 14/342 E2F sites overlapped at least partially with the GC-rich motifs of our bipartite DBSs, whereas 27/64 CHR motifs from the TGR database exhibited a partial overlap with the AT-rich motifs. Importantly, most E2F and CHR sites from the TGR database mapped close to E2F4 and LIN9 ChIP peaks, but only 16% of E2Fs (54/342), and 33% of CHRs (21/64) mapped precisely at the level of these peaks (Fig. S11), compared to 55% (83/151) of our bipartite DBSs (Fig. 3A). Thus, at least for genes with bipartite DBSs, our method relying on PFM22 appeared to provide more reliable predictions of DREAM binding than the E2F and CHR sites reported separately in the TGR database. Importantly, however, predictions of the TGR database might include genes regulated by a single E2F or a single CHR that would most likely remain undetected with PFM22, suggesting that both approaches provide complementary results. Of note, we previously used ConSite (Sandelin et al., 2004) with PFMs from six or eight experimentally demonstrated murine DBSs (Filipescu et al., 2017; Jaber et al., 2016) to search for bipartite DBSs, a method suitable for the analysis of small (≤10 kb) DNA sequences. Here, the use of PWMscan with PFMs from ten or 22 DBSs made it possible to perform genome-wide searches for bipartite DBSs, while facilitating the comparison of mouse and human DNA sequences. Our improved approach notably led to the identification of a functional DBS for *Fanca*, a gene we previously found to be downregulated by p53 but for which a DBS remained to be identified (Jaber et al., 2016; Simeonova et al., 2013).

Finding a functionally relevant DBS for *Fanca*, mutated in 60% of patients with Fanconi anemia (Balta et al., 2000; Fanconi anaemia/Breast cancer consortium, 1996), may help to understand how a germline increase in p53 activity can cause defects in DNA repair. Importantly, however, we previously showed that *p53^Δ31/Δ31^* cells exhibited defects in DNA interstrand cross-link repair, a typical property of Fanconi anemia cells, that correlated with a subtle but significant decrease in expression for several genes of the Fanconi anemia DNA repair pathway rather than the complete repression of a single gene in this pathway (Jaber et al., 2016). Thus, the Fanconi-like phenotype of *p53^Δ31/Δ31^* cells most likely results from a decreased expression of not only *Fanca*, but also additional p53-DREAM targets mutated in Fanconi anemia such as *Fancb*, *Fancd2*, *Fanci*, *Brip1*, *Rad51*, *Palb2*, *Ube2t* or *Xrcc2*, for which functional or putative DBSs were also found with our systematic approach. Furthermore, our identification of a DBS for *Rtel1*, a gene mutated in 30% of patients with Hoyeraal–Hreidarsson syndrome (Ballew et al., 2013; Deng et al., 2013; Le Guen et al., 2013; Walne et al., 2013) and the expression of which correlated with the survival of *p53^Δ31/Δ31^* mice (Simeonova et al., 2013), might explain how a germline increase in p53 activity can cause defects in telomere maintenance (Simeonova et al., 2013; Toufektchan et al., 2020). However, it remains possible that the p53-dependent repression of additional genes, such as *Dclre1b*, mutated in dyskeratosis congenita, or *Fancd2* (Joksic et al., 2012), might also affect telomere maintenance. Likewise, increased p53 activity was reported to partially phenocopy Diamond–Blackfan anemia, through mechanisms that remained unknown (Toki et al., 2018). Our finding that *Tsr2*, a gene mutated in Diamond–Blackfan anemia (Gripp et al., 2014), is repressed by p53 and DREAM provides a possible explanation for Diamond–Blackfan anemia-like phenotypes consecutive to germline p53 activation, but the p53-dependent repression of *Fanca* might also contribute to altered ribosome biogenesis (Gueiderikh et al., 2021). Taken together, these data suggest that increased p53 activity may cause bone marrow failure through several possible mechanisms by promoting the DREAM-mediated repression of many genes. Although this complexity may hamper the identification of the most clinically relevant p53-DREAM targets, it might also account for the partial phenotypic overlap between bone marrow failure syndromes of distinct molecular origins, as discussed previously (Jaber et al., 2016). Indeed, defects in telomere maintenance, DNA repair or ribosome function would all lead to p53 activation (Ceccaldi et al., 2012; Chin et al., 1999; McGowan et al., 2008), and the subsequent DREAM-mediated gene repression might have similar downstream consequences, leading to common clinical traits. Furthermore, our analyses indicated that many targets of the p53-DREAM pathway are associated with microcephaly or cerebellar hypoplasia, also suggesting that DREAM-mediated concomitant downregulation of multiple genes might contribute to these pathological processes. Consistent with this possibility, ZIKV is known to cause p53 activation in cortical neural progenitors and microcephaly (Tang et al., 2016; Zhang et al., 2016), and genetic analyses in ZIKV-infected mice indicated that variations in clinical severity and brain pathology between different mouse strains were driven by multiple host genes with small effects (Manet et al., 2020).

Our analysis suggests that many targets of the p53-DREAM pathway are associated with syndromes of abnormal hematopoiesis or brain development. To get a more precise evaluation of this association, we searched for genetic disorders that might be caused by the mutation of any of the 151 candidate p53-DREAM targets for which putative DBSs were identified. According to the Online Mendelian Inheritance in Man (OMIM) catalog, an online catalog of human genes and genetic disorders (https://www.omim.org), 106/151 genes were mutated in a hematological or neurological disorder. Among these, 25 were mutated in syndromes characterized by anemia, lymphopenia, neutropenia or thrombocytopenia and 77 in syndromes with microcephaly, cerebellar hypoplasia or hypoplasia of cerebellar vermis, including 13 associated with both types of symptoms (Table 3; Table S36). Among these 13 genes is, notably, *Nuf2*, mutations of which were initially shown to cause microcephaly (Uehara et al., 2021) but were later also associated with bone marrow failure (Vial et al., 2022). Furthermore, out of 317 potential DREAM targets, 58 were associated with both blood- and brain-related GO terms (Tables S17 and S26). This suggests that it might be worthwhile to systematically search for hematopoietic anomalies in patients with syndromes of abnormal brain development and, conversely, to check for neurological anomalies in patients with syndromes of abnormal hematopoiesis.

**Table 3.**
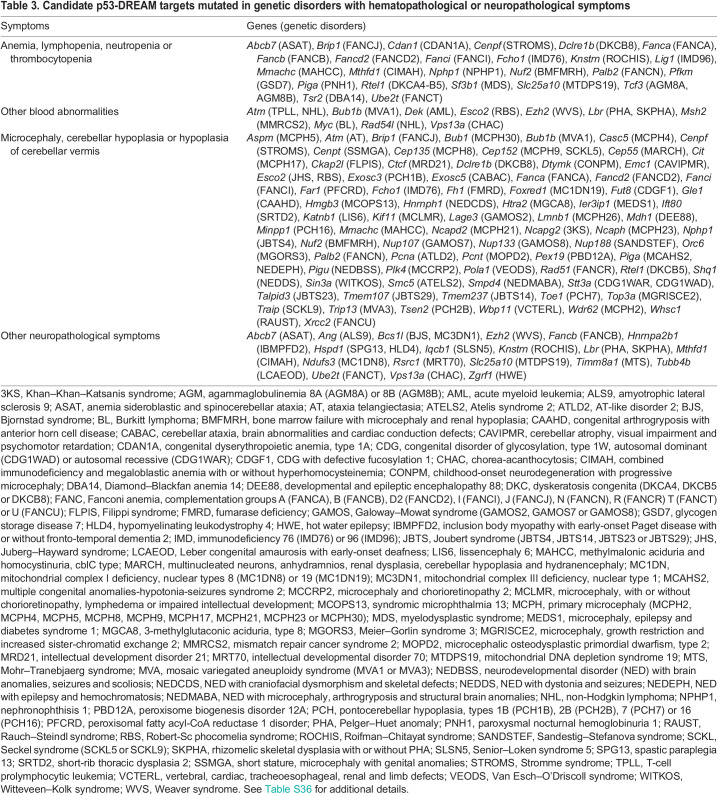
Candidate p53-DREAM targets mutated in genetic disorders with hematopathological or neuropathological symptoms

**Fig. 4.**
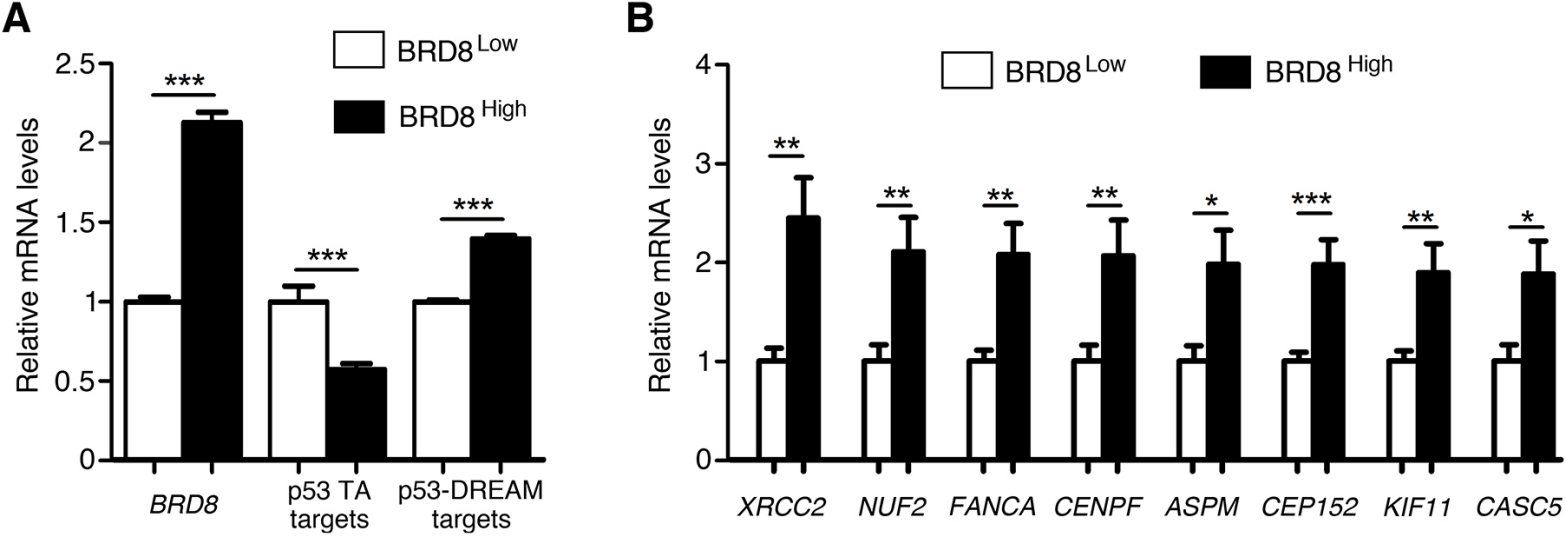
**In glioblastoma cells, high *BRD8* levels correlate with an increased expression of brain-related p53-DREAM targets.** (A) Glioblastoma cells with high *BRD8* levels exhibit an overall increased expression of brain-related p53-DREAM target genes. RNAseq data from glioblastoma cells isolated from patient specimens (GSE121720) were stratified according to *BRD8* mRNA levels as previously described (Sun et al., 2023) and, for each gene, the average expression levels in tumors expressing low *BRD8* levels was assigned a value of 1. The relative expression for *BRD8*, five p53-transactivated (TA) target genes (*CDKN1A*, *MDM2*, *BAX*, *GADD45A* and *PLK3*) and the 77 p53-DREAM targets associated with microcephaly or cerebellar hypoplasia (from Table 3) are shown. Tumors with high *BRD8* expression levels exhibit decreased expression levels of p53-transactivated targets and increased levels of p53-DREAM targets. Data are from 23 samples per group for *BRD8*, 115 values per group (23 samples×5 genes) for p53-transactivated targets and 1771 values per group (23 samples×77 genes) for p53-DREAM targets. (B) *CENPF*, *ASPM* and *CASC5* are among the eight p53-DREAM target genes for which their expression is most affected by *BRD8* levels in glioblastoma cells. Data for the indicated genes (with 23 samples per group) were retrieved from dataset GSE121720 and analyzed as in A. Data show the mean±s.e.m. *P<0.05; **P<0.01; ***P<0.001 (two-tailed unpaired Student's *t*-test). See Table S37 for additional details.

The p53-DREAM targets we identified are likely to be overexpressed in cells with mutant p53, a frequent alteration in cancer cells. For some p53-DREAM targets, such an overexpression may promote tumorigenesis. For example, TRIP13 was shown to promote cancer cell proliferation and the epithelial-mesenchymal transition in various tumor types (Dong et al., 2019; Li et al., 2021; Lu et al., 2022; Niu et al., 2019; Sheng et al., 2018; Zhou and Shu, 2019), and we identified here a functionally relevant DBS regulating *Trip13* expression. In cells with mutant p53, an increase in TRIP13 expression might thus be one of the mechanisms favoring the epithelial-mesenchymal transition. Importantly, many of the p53-DREAM targets we identified play a role in brain development, suggesting that the impact of a loss or attenuation of the p53-DREAM pathway might be particularly relevant for brain tumorigenesis. In support of this possibility, the chromatin regulator bromodomain-containing protein 8 (BRD8) was recently shown to attenuate p53 in glioblastoma (Sun et al., 2023), and we observed, in glioblastoma cells with high BRD8 levels (Wu et al., 2020), an overall increased expression for the 77 p53-DREAM targets associated with microcephaly or cerebellar hypoplasia (Fig. 4A; Table S37). Furthermore *CENPF*, *ASPM* and *CASC5* are known to contribute to phenotypic variation in glioblastoma neoplastic cells (Wang et al., 2022) and they were among the eight p53-DREAM target genes most affected by BRD8 levels (Fig. 4B; Table S37).

Altogether, this analysis expands our knowledge of the p53-DREAM pathway and notably indicates that this pathway regulates many genes implicated in bone marrow failure syndromes, neurodevelopmental disorders and cancer, suggesting an explanation for the variety of clinical symptoms that might result from its deregulation. Furthermore, our PFMs, which were useful in the identification of functionally relevant DBSs in genes associated with blood- or brain-related syndromes, should be considered to analyze the promoters of additional DREAM targets, implicated in other pathologies.

## MATERIALS AND METHODS

### Transcriptome data comparisons

We analyzed the gene expression data from Hoxa9-ER-expressing hematopoietic stem and progenitor cells grown in the presence of tamoxifen or in differentiated cells 5 days after tamoxifen withdrawal, a microarray study relying on 45,101 probes corresponding to 20,627 genes (Gene Expression Omnibus GSE21299; Muntean et al., 2010). For each probe, we calculated the inverse of log_2_ from robust multi-average values. The obtained average (from triplicates) for cells with tamoxifen was given a value of 1, and the ratios before and after tamoxifen withdrawal were calculated. For each gene, we took the probe leading to the highest repression ratio into account and selected those downregulated at least 1.5-fold upon tamoxifen withdrawal. Among these genes, we identified targets downregulated by human and/or mouse p53 by consulting p53 regulation scores in the TGR database (http://www.targetgenereg.org/) (Fischer et al., 2022a). Relative expression data were graphed with Microsoft Excel using a two-color scale and conditional coloring.

### GO analyses

To identify genes associated with bone marrow failure, we first used the GOrilla (Technion) software (Eden et al., 2009). Out of 20,627 genes analyzed by microarray, 17,461 were associated with a GO term according to GOrilla. A downregulation of at least 1.5-fold upon tamoxifen withdrawal was observed for 6880 probes corresponding to 4571 genes, of which 3631 were associated with a GO term. Enrichment analyses were carried out by comparing the unranked list of genes downregulated at least 1.5-fold (target) to the full list of genes (background), with ontology searches for biological processes or molecular function and default *P*-value settings (10^−3^). Independently, for both blood- and brain-related genes, we used the GO lists from the Human Phenotype Ontology website (Köhler et al., 2021).

### ChIP-seq data analyses

We used the peak browser from ChIP-Atlas (https://chip-atlas.org/peak_browser) (Zou et al., 2022) to search for E2F4 and LIN9 binding on the *Mus musculus* (mm10) genome or on the *Homo sapiens* (hg38) genome, and visualized results on the Integrative Genomics Viewer (IGV version 2.12.2) (Robinson et al., 2011). Peaks from all cell types were analyzed, and those with the highest binding score and minimal distance from the TSS were selected for. ChIP binding scores were graphed with Microsoft Excel using a two-color scale and conditional coloring.

### RNAseq data analyses

To screen for the most relevant candidate p53-DREAM targets, we analyzed the publicly available datasets GSE171697 and GSE204924 for genes with blood-related ontology terms, and the datasets GSE78711 and GSE80434 for genes with brain-related ontology terms. In addition, we analyzed the dataset GSE121720, containing RNAseq data from glioblastoma cells isolated from patient specimens. This dataset contains 92 samples, which were ranked according to *BRD8* expression levels, and the top and bottom 25% samples were assigned as the BRD8^high^ and BRD8^low^ groups, as previously described (Sun et al., 2023; Wu et al., 2020). Data for the 77 p53-DREAM targets associated with microcephaly or cerebellar hypoplasia (from Table 3) were then retrieved from the dataset and analyzed.

### Search for putative DBSs

To search for putative DBSs, we used PWMScan (https://epd.expasy.org/pwmtools/pwmtools/pwmscan.php; Ambrosini et al., 2018) with a custom PFM from 10, 21 or 22 murine functional DBSs (for details, see Fig. S7) on both the mouse (mm10) and human (hg38) genomes with a *P*-value threshold of 10^−3^. The putative DBSs identified were then analyzed for sequence conservation between mouse and human genomes and classified according to PFM scores and numbers of mismatches between the two species at positions 2-6 (for CDE or E2F) and 11-16 (for CHR or CLE) of the DBSs. For 151 genes, the identified putative DBSs, as well as putative E2F or CHR sites reported in the TGR database, were mapped relative to ChIP peaks (or TSSs) using the Integrative Genomics Viewer (IGV version 2.12.2).

### Cells and cell culture reagents

NIH3T3 cells or MEFs isolated from 13.5 days post-coitum embryos and cultured for <5 passages were cultured in a 5% CO_2_ and 3% O_2_ incubator, in Dulbecco's modified Eagle medium (DMEM) GlutaMAX (Gibco), with 15% fetal bovine serum (FBS; PAN-Biotech), 100 µM 2-mercaptoethanol (Millipore), 0.01 mM non-essential amino-acids and penicillin/streptomycin (Gibco). Cells were treated for 24 h with 10 µM Nutlin 3a (Sigma-Aldrich).

### Quantitative real-time PCR

Total RNA was extracted using nucleospin RNA II (Macherey-Nagel), reverse-transcribed using superscript IV (Invitrogen), and real-time quantitative PCRs were performed on an ABI PRISM 7500 using Power SYBR Green (Applied Biosystems) as previously described (Simeonova et al., 2013). Primer sequences are listed in Table S38.

### Luciferase assays

For each tested gene, a 1-1.5 kb fragment of the promoter containing the putative DBS at its center was cloned upstream of a luciferase reporter gene in the backbone of a PGL3 basic vector (Promega). For all tested DBSs, to prevent DREAM binding, we used PCR mutagenesis and mutated the putative binding site into the following sequence: 5′-AAATAA(NNNN)AGACTG-3′, with ‘(NNNN)’ corresponds to DNA spacer sequences that were not mutated. We used Lipofectamine 2000 (Invitrogen) to transfect ∼10^6^ NIH3T3 cells with 3 µg of the luciferase plasmid with a WT DBS or its mutant counterpart and 30 ng of a Renilla luciferase expression plasmid (pGL4.73, Promega) for normalization, and treated with or without 10 µM Nutlin 3a. The transfected cells were incubated for 24 h, then trypsinized, resuspended in 75 µl culture medium with 7.5% FBS and transferred into wells of an optical 96-well plate (Nunc). The Dual-Glo luciferase assay system (Promega) was used according to the manufacturer's protocol to lyse the cells and read Firefly and Renilla luciferase signals. Results were normalized, then the average luciferase activity in untreated cells transfected with a WT promoter were assigned a value of 1.

### Statistical analyses

Two-tailed unpaired Student's *t*-tests were used to analyze differences between undifferentiated and differentiated BMCs, between WT and *p53^Δ31/Δ31^* BMCs, between WT or mutant promoters in luciferase assays, and between glioblastomas with low and high BRD8 levels. Two-tailed paired Student's *t*-tests were used to analyze differences between untreated and Nutlin-treated cells. Analyses were performed using GraphPad Prism 5, and values of *P*<0.05 were considered significant. Hypergeometric testing of DBS distributions was performed with the Keisan calculator (https://keisan.casio.com/).

## Supplementary Material

10.1242/dmm.050376_sup1 Supplementary information

## First Person

This article has an associated First Person interview with the first author of the paper.
