# Supplementary material for: A systematic approach identifies p53-DREAM pathway target genes associated with blood or brain abnormalities
Source: Dis Model Mech. 2023 Oct 11;16(10):dmm050376. doi: 10.1242/dmm.050376 (PMC10581385; doi:10.1242/dmm.050376)
Supplement: Supplementary information [file dmm-16-050376-s1.pdf]

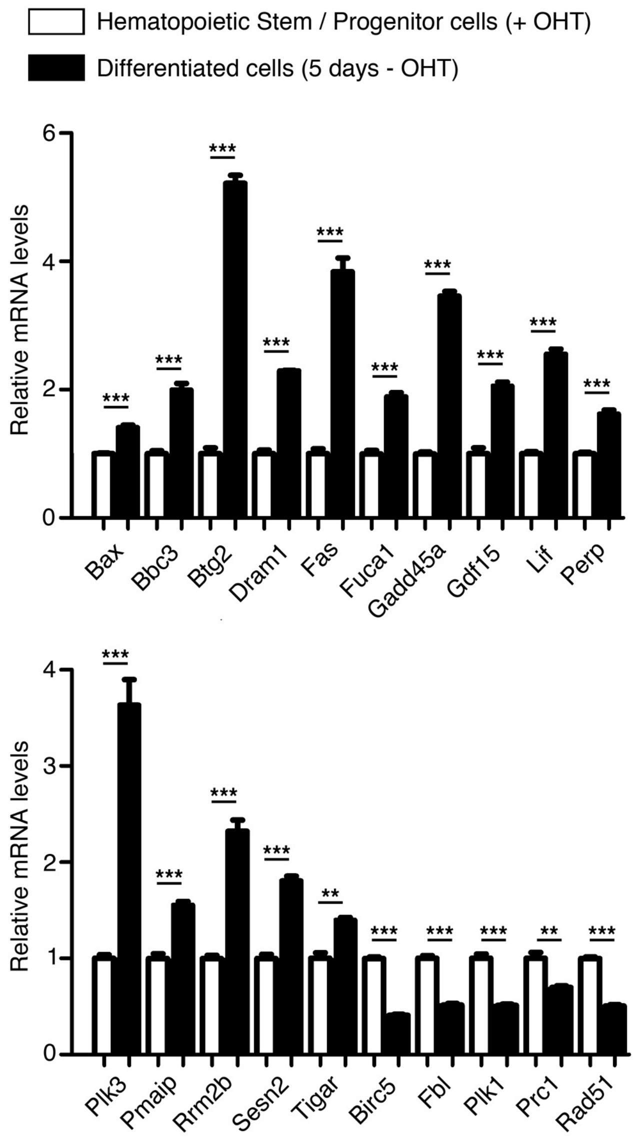

Fig. S1. Additional evidence that Hoxa9-ER expressing bone marrow cell differentiation correlates with p53 activation. Robust-multi average values for expression of the indicated genes were extracted from transcriptome data of Hoxa9-ER expressing hematopoietic stem and progenitor cells (grown in the presence of tamoxifen) or differentiated cells (5 days after tamoxifen withdrawal). Average values (from triplicates) in cells with tamoxifen were given a value of 1. Upon p53 activation, *Bax*, *Bbc3/Puma*, *Btg2*, *Dram1*, *Fas*, *Fuca1*, *Gadd45a*, *Gdf15*, *Lif*, *Perp*, *Plk3*, *Pmaip*, *Rrm2b*, *Sesn2* and *Tigar* are known to be transactivated, whereas *Birc5/Survivin*, *Fbl*, *Plk1*, *Prc1* and *Rad51* are known to be downregulated. Means + s.e.m. are shown; ***$P<0.001$, **$P<0.01$ by Student's t test.

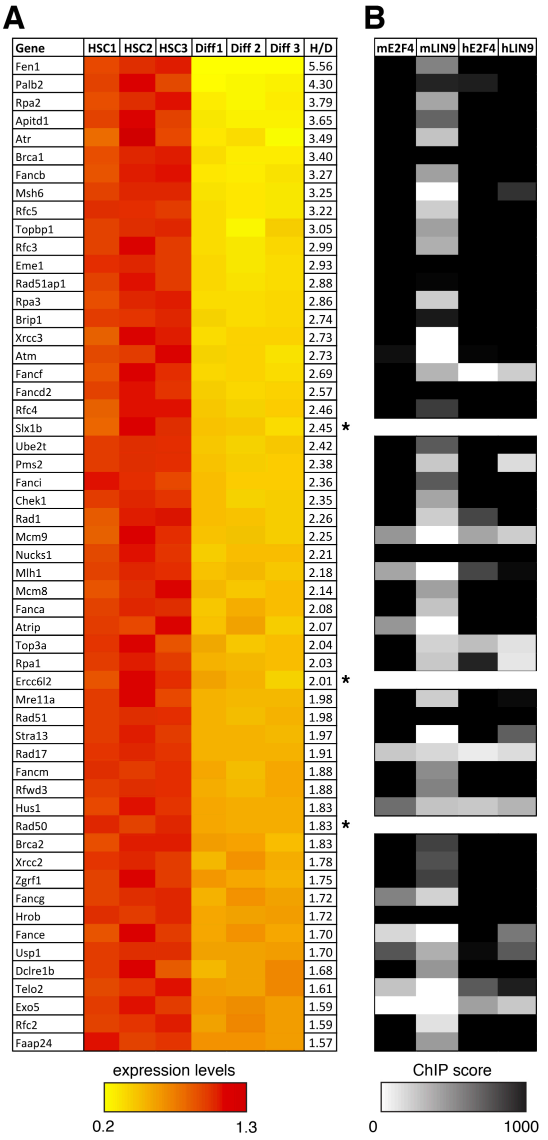

Fig. S2. Fanconi-related genes downregulated upon bone marrow cell differentiation, and their potential regulation by DREAM. (A) Expression values, represented as described in Fig. 1B, for 55 genes related to the Fanconi anemia DNA repair pathway, of which 52 are downregulated by p53. (B) ChIP scores of E2F4 or LIN9 binding in mouse (m) or human (h) cells for the 52 Fanconi-related, p53-regulated genes, represented as in Fig. 1C.

Disease Models & Mechanisms • Supplementary information

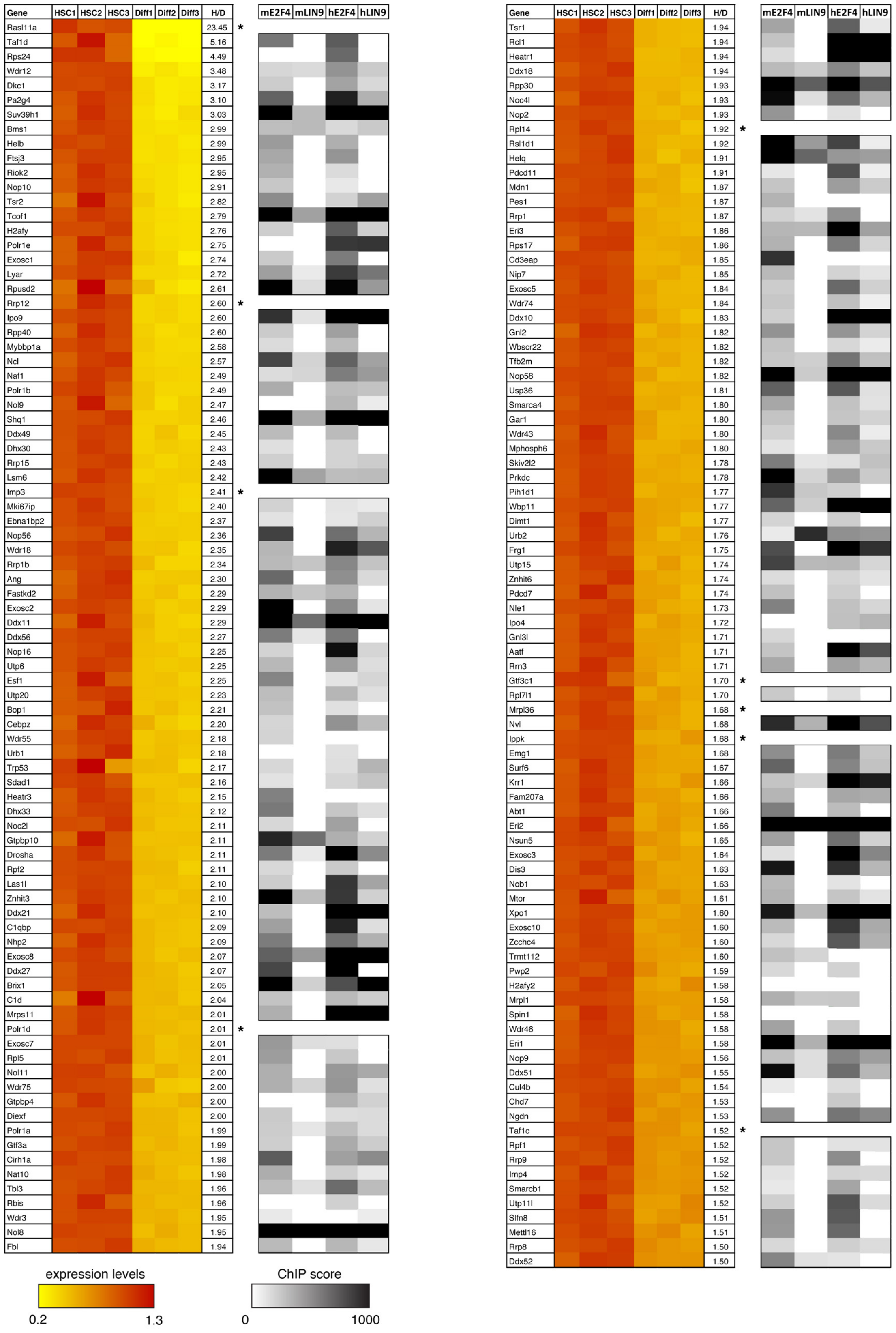

Fig. S3. Ribosome-related genes downregulated upon bone marrow cell differentiation, and their potential regulation by DREAM. Expression values and highest E2F4 and LIN9 ChIP binding scores for 171 ribosome-related genes, represented as described in Fig. 1B-C.

Disease Models & Mechanisms • Supplementary information

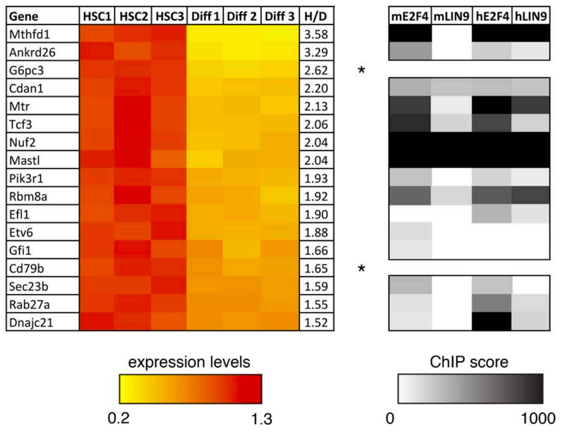

Fig. S4. Other genes associated with bone marrow failure syndromes, downregulated upon bone marrow cell differentiation, and their potential regulation by DREAM. Expression values and highest E2F4 and LIN9 ChIP binding scores for 17 genes mutated in other bone marrow failure syndromes, represented as described in Fig. 1B-C.

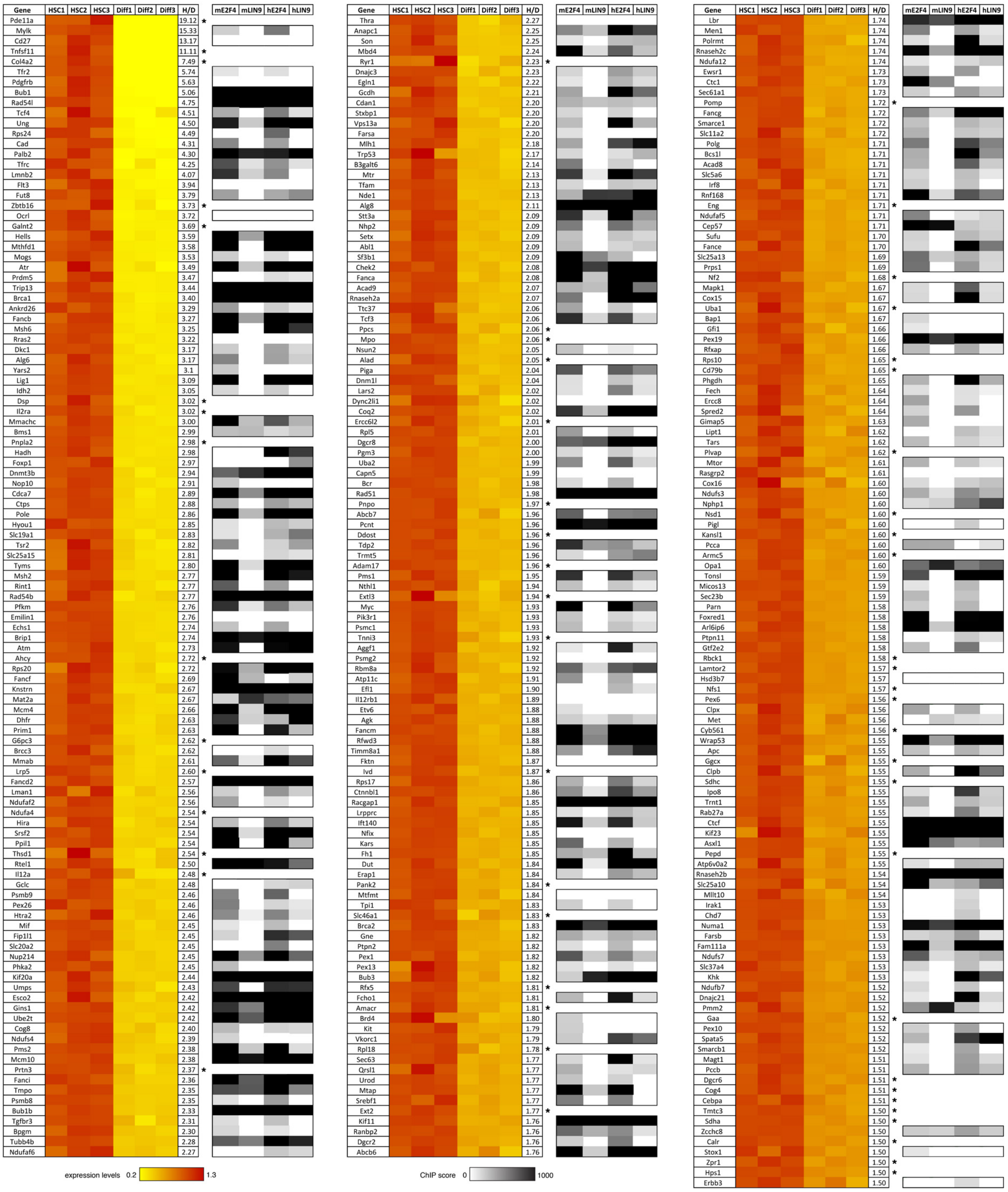

Fig. S5. Genes associated with abnormalities in blood or blood-forming tissues and downregulated upon bone marrow cell differentiation, and their potential regulation by DREAM. Expression values and highest E2F4 and LIN9 ChIP binding scores for 336 genes associated with abnormalities in blood and blood-forming tissues (according to the Human Phenotype Ontology website of the Jackson laboratory, ontology term #HP:001871), represented as described in Figure 1B-C.

Disease Models & Mechanisms • Supplementary information

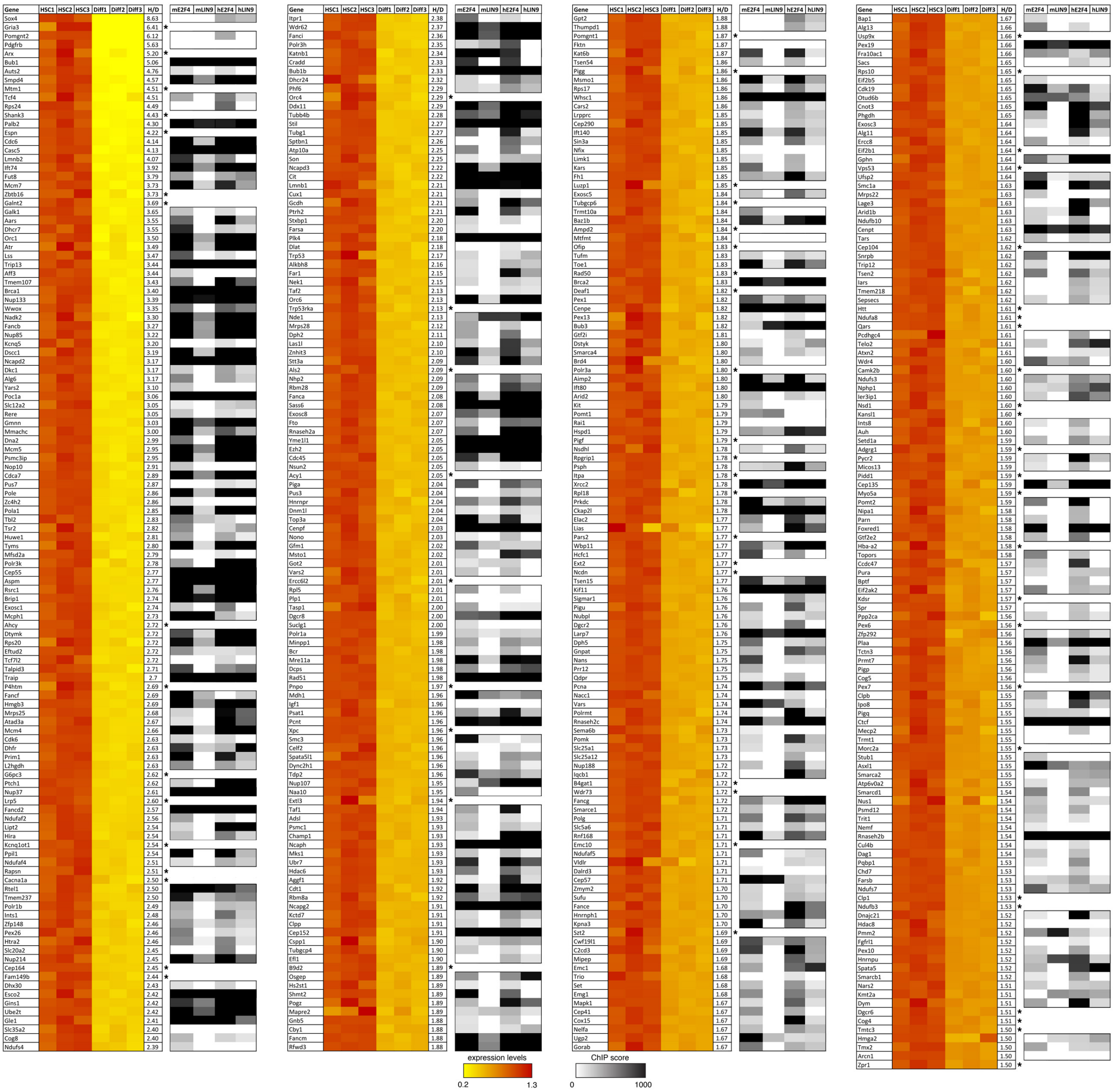

Fig. S6. Genes associated with microcephaly or cerebellar hypoplasia and downregulated upon bone marrow cell differentiation, and their potential regulation by DREAM. Expression values and highest E2F4 and LIN9 ChIP binding scores for 474 genes associated with microcephaly or cerebellar hypoplasia (according to the Human Phenotype Ontology website of the Jackson laboratory, ontology terms #HP0000252 and HP:0007360), represented as described in Figure 1B-C.

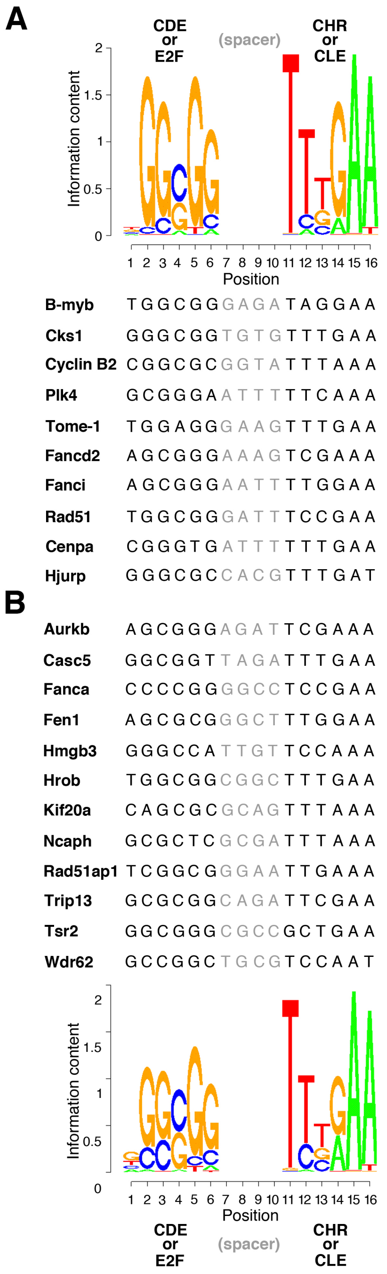

Fig. S7. Positional frequency matrices used to search for DREAM binding sites. (A) PFM10, a positional frequency matrix from 10 experimentally validated DREAM binding sites (DBS). The logo for PFM10 (top) and DNA sequences from the 10 experimentally validated murine DBS used to generate it (bottom) are shown. Spacer DNA sequences between GC-rich (CDE or E2F) and AT-rich (CHR or CLE) elements were not used to define the matrix. (B) PFM22, a positional frequency matrix from 22 experimentally tested DBS. The DNA sequences of 12 additional murine DBS tested in this study (top) were added to the first 10 DBS to define PFM22 (bottom). Spacer DNA sequences between GC-rich (CDE or E2F) and AT-rich (CHR or CLE) elements were not taken into account to define the matrix. Compared to PFM10, PFM22 notably introduces minor nucleotides at positions 2 (A), 5 (C), 6 (T) and 11 (G), while decreasing the frequency of rare nucleotides at positions 4 (A) and 12 (A).

Disease Models & Mechanisms • Supplementary information

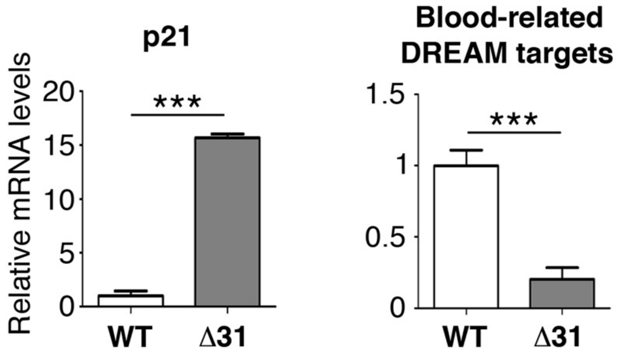

Fig. S8. Bone marrow cells from p53$^{\Delta31/\Delta31}$ mice exhibit increased p21 gene expression, and a decreased expression of blood-related candidate p53-DREAM targets. mRNAs extracted from the bone marrow cells of wild-type (WT) and p53$^{\Delta31/\Delta31}$ (D31) mice were quantified using real-time PCR, normalized to control mRNAs, then the amount in WT untreated cells was assigned a value of 1. The blood–related p53-DREAM target genes tested are the 8 genes reported in Fig. 2A-C : *Aurkb*, *Fanca*, *Fen1*, *Hrob*, *Kif20a*, *Rad51ap1*, *Trip13* and *Tsr2*. Results from 2-3 mice per genotype, thus 16-24 values per group of DREAM target genes. ***P<0.001 by Student's t test.

Disease Models & Mechanisms • Supplementary information

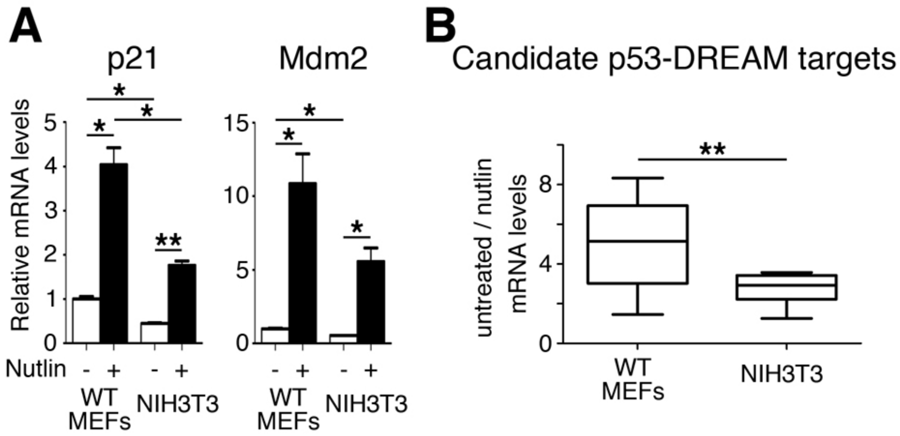

Fig. S9. The p53-DREAM pathway is attenuated in NIH3T3 cells. (A) The transactivation of p53 target genes is decreased in NIH3T3 cells. mRNAs from primary wild-type (WT) MEFs or NIH3T3 cells, untreated or treated with 10 mM Nutlin for 24h, were quantified using real-time PCR, normalized to control mRNAs, then the amount in WT untreated MEFs was assigned a value of 1. Means + s.e.m. from 2-3 independent experiments are shown. (B) Upon p53 activation, the repression of DREAM targets is less pronounced in NIH3T3 cells. The mRNAs for 12 candidate p53-DREAM targets (*Aurkb*, *Casc5*, *Fanca*, *Fen1*, *Hmgb3*, *Hrob*, *Kif20a*, *Ncaph*, *Rad51ap1*, *Trip13*, *Tsr2*, *Wdr62*), extracted from primary wild-type (WT) MEFs or NIH3T3 cells, untreated or treated with 10 mM Nutlin for 24h, were quantified using real-time PCR and normalized to control mRNAs as above, and ratios of nutlin-induced repression were calculated. For each tested gene, results are from 2-3 independent experiments. **P<0.01, *P<0.051 by Student's t test.

Disease Models & Mechanisms • Supplementary information

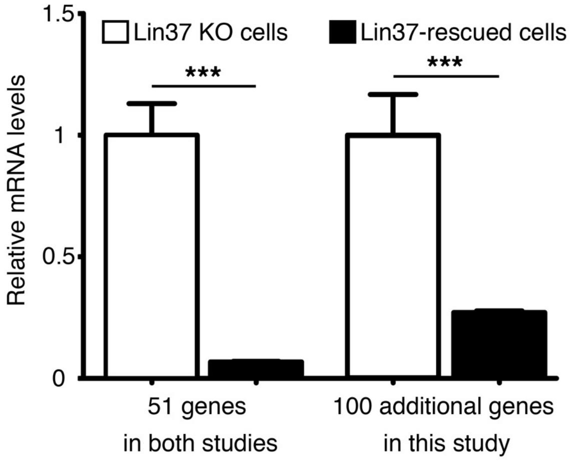

Fig. S10. The expression of candidate p53-DREAM targets is decreased in Lin37-rescued cells. For the 151 genes with putative DREAM binding sites (see Table 2), we extracted RNAseq data from dataset GSE97716, in LIN37 KO cells and LIN37-rescued cells. Data were from 2 different KO clones and 2 rescued clones, with 2-3 values per clone for each gene. For each gene, average expression values were calculated and a value of 1 was attributed for expression levels in LIN37 KO cells. Out of 151 genes, only 51 genes were previously reported to be DREAM targets based on their differential expression upon Lin37 reintroduction (for details, see main text and Table S35). Nevertheless, the other 100 genes were also downregulated upon Lin37 reintroduction, albeit with a smaller fold decrease. ***$P<0.001$ by Student's t test.

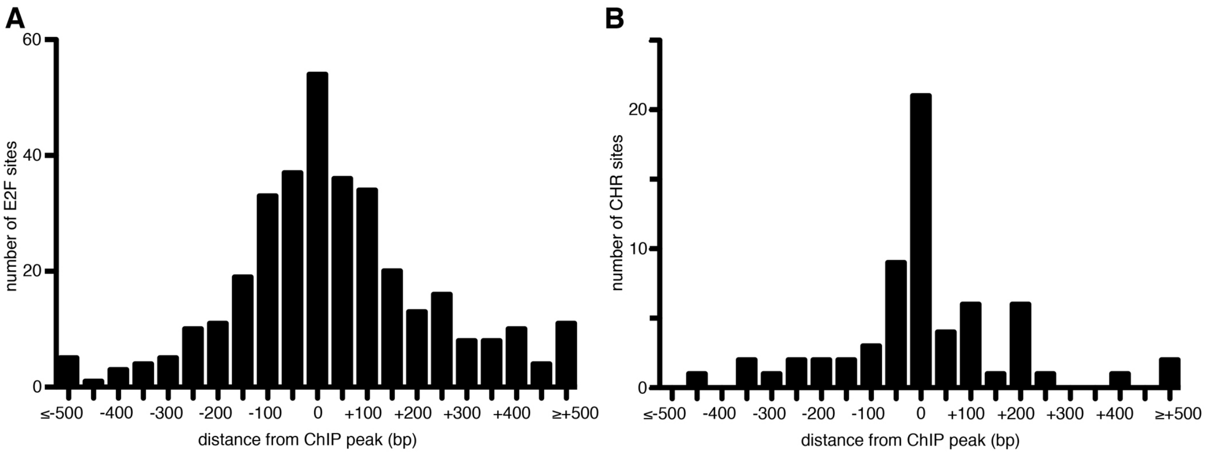

Fig. S11. Mapping of E2F and CHR motifs from the TGR database relative to ChIP peaks for DREAM subunits. (A) The 342 E2Fs motifs reported in the TGR database for the 151 genes listed in Table 2 were mapped relative to the ChIP peaks of E2F4 and/or LIN9 binding, in 50 bp windows. (B) The 64 CHR motifs reported in the TGR database for the 151 genes listed in Table 2 were mapped relative to the ChIP peaks of E2F4 and/or LIN9 binding, in 50 bp windows.

Table S1. Genes implicated in telomere biology and downregulated upon HSC differentiation, and their potential regulation by p53.

Expression values from triplicates were retrieved from dataset #GSE21299. Genes are listed according to decreasing repression fold. Scores from the Target gene regulation (TGR) database indicate p53-regulated genes, with negative p53 TGR scores for repressed genes. Genes with a negative p53 TGR score in at least one species are highlighted in bold type.

Click here to download Table S1

Table S2. E2F4 and/or LIN9 binding relative to the transcription start site (TSS) of murine genes implicated in telomere biology and downregulated upon HSC differentiation.

Genes with a negative p53 TGR score in at least one species (Table S1) were analyzed, and ordered as in Figure 1B. ChIP scores and positions are from ChIP-Atlas, M. musculus mm10.

Click here to download Table S2

Table S3. E2F4 and/or LIN9 binding relative to the transcription start site (TSS) of human genes homologous to the murine telomere-related genes listed in Table S2.

Genes are ordered as in Figure 1B. ChIP scores and positions are from ChIP-Atlas, H. sapiens hg38.

Click here to download Table S3

Table S4. Genes from GO terms related to the Fanconi anemia DNA repair pathway or to ribosome biology are over-represented among the genes down-regulated upon HSC differentiation.

GOrilla results are presented as in Table 1, for GO terms related to the Fanconi anemia DNA repair pathway (A) or ribosome biology (B).

Click here to download Table S4

Table S5. Genes implicated in the Fanconi Anemia pathway and downregulated upon HSC differentiation, and their potential regulation by p53.

Expression values from triplicates were retrieved from dataset #GSE21299. Genes are listed according to decreasing repression fold. Scores from the Target gene regulation (TGR) database indicate p53-regulated genes, with negative p53 TGR scores for repressed genes.

Genes with a negative p53 TGR score in at least one species are highlighted in bold type.

Click here to download Table S5

Table S6. E2F4 and/or LIN9 binding relative to the TSS of murine genes implicated in the Fanconi Anemia pathway and downregulated upon HSC differentiation.

Genes with a negative p53 TGR score in at least one species (Table S5) were analyzed, and ordered as in Figure S2. ChIP scores and positions are from ChIP-Atlas, M. musculus mm10.

Click here to download Table S6

Disease Models & Mechanisms • Supplementary information

Table S7. E2F4 and/or LIN9 binding relative to the TSS of human genes homologous to the murine Fanconi-related genes listed in Table S6.

Genes are ordered as in Figure S2. ChIP scores and positions are from ChIP-Atlas, H. sapiens hg38.

Click here to download Table S7

Table S8. Genes implicated in ribosome biology and downregulated upon HSC differentiation, and their potential regulation by p53.

Expression values from triplicates were retrieved from dataset #GSE21299. Genes are listed according to decreasing repression fold. Scores from the Target gene regulation (TGR) database indicate p53-regulated genes, with negative p53 TGR scores for repressed genes. Genes with a negative p53 TGR score in at least one species are highlighted in bold type.

Click here to download Table S8

Table S9. E2F4 and/or LIN9 binding relative to the TSS of murine genes implicated in ribosome biology and downregulated upon HSC differentiation.

Genes with a negative p53 TGR score in at least one species (Table S8) were analyzed, and ordered as in Figure S3. ChIP scores and positions are from ChIP-Atlas, M. musculus mm10.

Click here to download Table S9

Table S10. E2F4 and/or LIN9 binding relative to the TSS of human genes homologous to the murine ribosome-related genes listed in Table S9.

Genes are ordered as in Figure S3. ChIP scores and positions are from ChIP-Atlas, H. sapiens hg38.

Click here to download Table S10

Table S11. Genes implicated in rare bone marrow failure syndromes and downregulated upon HSC differentiation, and their potential regulation by p53.

Expression values from triplicates were retrieved from dataset #GSE21299. Genes are listed according to decreasing repression fold. Scores from the Target gene regulation (TGR) database indicate p53-regulated genes, with negative p53 TGR scores for repressed genes.

Genes with a negative p53 TGR score in at least one species are highlighted in bold type.

Click here to download Table S11

Table S12. E2F4 and/or LIN9 binding relative to the TSS of murine genes implicated in rare bone marrow failure syndromes and downregulated upon HSC differentiation.

Genes with a negative p53 TGR score in at least one species (Table S11) were analyzed, and ordered as in Figure S4. ChIP scores and positions are from ChIP-Atlas, M. musculus mm10.

Click here to download Table S12

Disease Models & Mechanisms • Supplementary information

Table S13. E2F4 and/or LIN9 binding relative to the TSS of human homologs to the murine genes implicated in rare bone marrow failure syndromes listed in Table S12.

Genes are ordered as in Figure S4. ChIP scores and positions are from ChIP-Atlas, H. sapiens hg38.

Click here to download Table S13

Table S14. Genes associated with abnormalities in blood and blood-forming tissues and downregulated upon HSC differentiation, and their potential regulation by p53.

Genes are associated with abnormalities in blood and blood-forming tissues according to Human Phenotype Ontology (#HP:0001871).

Expression values from triplicates were retrieved from dataset #GSE21299. Genes are listed according to decreasing repression fold. Scores from the Target gene regulation (TGR) database indicate p53-regulated genes, with negative p53 TGR scores for repressed genes.

Genes with a negative p53 TGR score in at least one species are highlighted in bold type.

Click here to download Table S14

Disease Models & Mechanisms • Supplementary information

Table S15. E2F4 and/or LIN9 binding for mouse genes downregulated upon HSC differentiation and associated with abnormalities in blood and blood-forming tissues according to Human Phenotype Ontology (#HP:0001871).
Genes with a negative p53 TGR score in at least one species (Table S14) were analyzed, and ordered as in Figure S5. ChIP scores and positions are from ChIP-Atlas, M. musculus, mm10.

Click here to download Table S15

Table S16. E2F4 and/or LIN9 binding relative to the TSS of human homologs to the murine genes associated with abnormalities in blood and blood-forming tissues listed in Table S15.
Genes are ordered as in Figure S5. ChIP scores and positions are from ChIP-Atlas, H. sapiens hg38.

Click here to download Table S16

Table S17. A summary of our approach to select blood ontology-related candidate p53-DREAM targets.
see text for details.

Click here to download Table S17

Disease Models & Mechanisms • Supplementary information

Table S18. A summary of blood-related genes downregulated upon BMC differentiation, highlighting those with DNA sequences bound by both E2F4 and LIN9 in at least one species.

571 genes associated with blood abnormalities were downregulated upon BMC differentiation, including 499 reported to be downregulated by p53. E2F4 and LIN9 bound similar regions for 374 genes, for which the total ChIP scores are indicated. The 269 genes with a score ≥ 979 are highlighted in bold type and correspond to the best candidate DREAM targets. Genes are ordered alphabetically in each category.

Click here to download Table S18

Table S19. Expression of p53 transactivated genes and candidate p53-DREAM blood-related genes in datasets GSE171697 and GSE204924.

Genes are ordered alphabetically in each category. Out of 269 candidate p53-DREAM targets, 56 appeared upregulated in cells with increased p53 activity (average ratio (avg R) ≥ 1): these genes were considered poor candidates and removed from further studies. The other 213 candidate genes are noted in bold.

Click here to download Table S19

Disease Models & Mechanisms • Supplementary information

Table S20. Genes mutated in syndromes of microcephaly or cerebellar hypoplasia and downregulated upon HSC differentiation, and their potential regulation by p53.

Expression values from triplicates were retrieved from dataset #GSE21299. Genes are listed according to decreasing repression fold. Scores from the Target gene regulation (TGR) database indicate p53-regulated genes, with negative p53 TGR scores for repressed genes. Genes with a negative p53 TGR score in at least one species are highlighted in bold type.

Click here to download Table S20

Table S21. E2F4 and/or LIN9 binding for murine genes downregulated upon HSC differentiation that encode proteins mutated in syndromes with microcephaly or ponto-cerebellar hypoplasia.

Genes with a negative p53 TGR score in at least one species (Table S20) were analyzed, and ordered as in Figure 1D. ChIP maximum scores and positions are from ChIP-Atlas, M. musculus mm10.

Click here to download Table S21

Table S22. E2F4 and/or LIN9 binding for human genes homologous to the microcephaly- or cerebellar hypoplasia- related genes listed in Table S21.

Genes are ordered as in Figure 1D. ChIP maximum scores and positions are from ChIP-Atlas, H. sapiens hg38.

Click here to download Table S22

Table S23. Genes implicated in microcephaly or cerebellar hypoplasia and downregulated upon HSC differentiation, and their potential regulation by p53.

Genes are associated with microcephaly or cerebellar hypoplasia according to Human Phenotype Ontology (#HP:0000252, 0007360). Expression values from triplicates were retrieved from dataset #GSE21299. Genes are listed according to decreasing repression fold. Scores from the Target gene regulation (TGR) database indicate p53-regulated genes, with negative p53 TGR scores for repressed genes. Genes with a negative p53 TGR score in at least one species are highlighted in bold type.

Click here to download Table S23

Table S24. E2F4 and/or LIN9 binding for murine genes downregulated upon HSC differentiation and implicated in microcephaly or cerebellar hypoplasia according to Human Phenotype Ontology (#HP:0000252, 0007360).

Genes with a negative p53 TGR score in at least one species (Table S23) were analyzed, and ordered as in Figure S6. ChIP maximum scores and positions are from ChIP-Atlas, M. musculus mm10.

Click here to download Table S24

Table S25. E2F4 and/or LIN9 binding for human genes homologous to the microcephaly- or cerebellar hypoplasia- related genes listed in Table S25.

Genes are ordered as in Figure S6. ChIP maximum scores and positions are from ChIP-Atlas, H. sapiens hg38.

Click here to download Table S25

Disease Models & Mechanisms • Supplementary information

Table S26. A summary of our approach to select brain ontology-related candidate p53-DREAM targets.
see text for details.

Click here to download Table S26

Table S27. A summary of brain-related genes downregulated upon BMC differentiation, highlighting those with DNA sequences bound by both E2F4 and LIN9 in at least one species.
478 genes associated with brain abnormalities were downregulated upon BMC differentiation, including 408 reported to be downregulated by p53. E2F4 and LIN9 bound similar regions for 303 genes, for which the total ChIP scores are indicated. The 226 genes with a score ≥ 979 are highlighted in bold type and correspond to the best candidate DREAM targets. Genes are ordred alphabetically in each category.

Click here to download Table S27

Table S28. Expression of p53 transactivated genes and candidate p53-DREAM brain-related genes in datasets GSE78711 and GSE80434.
Genes are ordered alphabetically in each category. Out of 226 candidate p53-DREAM targets, 64 appeared upregulated after ZIKV infection (average ratio (avg R) ≥ 1): these genes were considered poor candidates and removed from further studies. The other 162 candidate genes are noted in bold.

Click here to download Table S28

Table S29. Genes involved in hematopoiesis with conserved putative DREAM binding sites.

All the putative DREAM binding sites (DBS) are in the same orientation as the transcribed putative target. M/H designate mouse or human sites, respectively. For each candidate, the DBS with higher PFM score is in upper case letters and mismatches in its homolog are in lower case. Putative DBS with a positive score calculated by PWMScan with the PFM10 are in bold. *for mismatch count, only mismatches at positions 2-6 and 11-16 of the DBS consensus were counted. **DBS conservation in ORF may result from selection of coding sequences rather than functional DBS. A combination of PFM score and degree of conservation was used to classify putative DBS in 4 categories (A-D), with A corresponding to the better candidates.

Click here to download Table S29

Table S30. Genes involved in microcephaly or cerebellar hypoplasia with conserved putative DREAM binding sites.

All the putative DREAM binding sites (DBS) are in the same orientation as the transcribed putative target. M/H designate mouse or human sites, respectively. For each candidate, the DBS with best PFM score is in upper case letters and mismatches in its homolog are in lower case (*for mismatch count. only mismatches at positions 2-6 and 11-16 of the DBS consensus were considered). Putative DBS with a positive score calculated by PWMScan with the PFM are in bold. A combination of PFM score and degree of conservation was used to classify putative DBS in 4 categories (A-D), with A corresponding to the better candidates.

Click here to download Table S30

Disease Models & Mechanisms • Supplementary information

Table S31. Genes involved in hematopoiesis with conserved putative DBS.

Genes for which a putative DBS were found with PFM10 (Table S29) were reanalyzed with PFM22. M/H designate mouse or human sites, respectively. All the putative DBS are in the same orientation as the transcribed putative target. For each candidate, the DBS with higher PFM score is in upper case letters and mismatches in its homolog are in lower case. Putative DBS with a positive score calculated by PWMScan with the PFM22 are in bold. *for mismatch count, only mismatches at positions 2-6 and 11-16 of the DBS consensus were counted. A combination of PFM score and degree of conservation was used to classify putative DBS in 4 categories (A-D) , with A corresponding to the better candidates.

Click here to download Table S31

Table S32. Genes involved in microcephaly or cerebellar hypoplasia with conserved putative DBS.

Genes for which a putative DBS was found with PFM10 (Table S30) were reanalyzed with PFM22. M/H designate mouse or human sites, respectively. All the putative DBS are in the same orientation as the transcribed putative target. For each candidate, the DBS with higher PFM score is in upper case letters and mismatches in its homolog are in lower case. Putative DBS with a positive score calculated by PWMScan with the PFM22 are in bold. *for mismatch count, only mismatches at positions 2-6 and 11-16 of the DBS consensus were counted. A combination of PFM score and degree of conservation was used to classify putative DBS in 4 categories (A-D), with A corresponding to the better candidates.

Click here to download Table S32

Table S33. Genes with putative DBS found only with PFM22.

Genes with E2F4 and LIN9 binding for which no putative DBS was found with PFM10 were analyzed with PFM22. M/H designate mouse or human sites, respectively. All the putative DBS are in the same orientation as the transcribed putative target. For each candidate, the DBS with higher PFM score is in upper case letters and mismatches in its homolog are in lower case. Putative DBS with a positive score calculated by PWMScan with the PFM22 are in bold. *for mismatch count, only mismatches at positions 2-6 and 11-16 of the DBS consensus were counted. A combination of PFM score and degree of conservation was used to classify putative DBS in 4 categories (A-D), with A corresponding to the better candidates.

Click here to download Table S33

Table S34. Genes with partially conserved putative DBS.

Genes with E2F4 and LIN9 binding were analyzed with PFM22. M/H designate mouse or human sites, respectively. All the putative DBS are in the same orientation as the transcribed putative target. For each candidate, the DBS with higher PFM score is in upper case letters and mismatches in its homolog are in lower case. Putative DBS with a positive score calculated by PWMScan with the PFM22 are in bold. *the DBS listed here display a perfect conservation of either the CDE/E2F (positions 2-6) or the Chr/CLE (positions 11-16).

Click here to download Table S34

Disease Models & Mechanisms • Supplementary information

Table S35. Expression of 151 p53-DREAM targets in Lin37 KO and Lin37-rescued murine cells.

For the 151 genes with putative DREAM binding sites (see Table 2), we extracted RNAseq data from dataset GSE97716. The 51 genes reported by Mages et al. to be differentially expressed upon Lin37 reintroduction are noted in bold.

Click here to download Table S35

Table S36. Candidate p53-DREAM targets mutated in blood or brain genetic disorders.

A,L,N,T: anemia, lymphopenia, neutropenia or thrombocytopenia ; OBA : other blood abnormalities; M, CH, HCV: microcephaly, cerebellar hypoplasia or hypoplasia of the cerebellar vermis; ONS: other neuropathological symptoms

Click here to download Table S36

Table S37. The expression of p53-DREAM targets associated with neurological disorders is increased in glioblastoma cells with high BRD8 levels.

RNAseq data for *BRD8*, five genes transactivated by p53 (*CDKN1A, BAX, GADD45A, MDM2, PLK3*) and the 77 p53-DREAM targets associated with microcephaly or cerebellar hypoplasia (listed in Table 3) were extracted from dataset GSE121720. The 23 glioblastoma samples with low expression levels of BRD8 (BRD8Low) are listed in bold type, the 23 samples with high BRD8 expression levels (BRD8High) are listed in bold italic type.

Click here to download Table S37

Disease Models & Mechanisms • Supplementary information

Table S38. DNA sequences of primers used in RTqPCR experiments.

For normalizations, primers for Ppia and Rplp0 were used in fibroblasts, and for Rplp0 and Rplp32 in bone marrow cells.

Click here to download Table S38
